# Burden of *Clostridioides difficile* infection (CDI) - a systematic review of the epidemiology of primary and recurrent CDI

**DOI:** 10.1186/s12879-021-06147-y

**Published:** 2021-05-19

**Authors:** Elaine Finn, Fredrik L. Andersson, Matthew Madin-Warburton

**Affiliations:** 1 IQVIA, 210 Pentonville Road, London, N1 9JY UK; 2 Ferring Pharmaceuticals, Copenhagen, Denmark

**Keywords:** *Clostridioides difficile*, Recurrent *Clostridioides difficile*, Epidemiology, Risk factors, Antibiotic resistance, Antimicrobial stewardship

## Abstract

**Background:**

*Clostridioides difficile* is a Gram-positive anaerobic bacterium, which causes *Clostridioides difficile* infection (CDI). It has been recognised as a leading cause of healthcare-associated infections and a considerable threat to public health globally. This systematic literature review (SLR) summarises the current evidence on the epidemiology and clinical burden of CDI.

**Methods:**

A SLR was conducted to identify CDI and recurrent CDI (rCDI) epidemiology studies, to evaluate patient and disease characteristics, incidence rates, epidemiological findings and risk factors. Embase, MEDLINE and the Cochrane Library databases were searched for English articles from 2009 to 2019. Included territories were the United Kingdom, France, Germany, Italy, Spain, Poland, US, Canada, Australia, Japan and China.

**Results:**

Of 11,243 studies identified, 165 fulfilled the selection criteria. An additional 20 studies were identified through targeted review of grey literature. The most widely reported findings were incidence and risk factors for CDI and rCDI.

Among key studies reporting both healthcare-associated (HA-CDI) and community-associated CDI (CA-CDI) incidence rates for each country of interest, incidence rates per 10,000 patient days in the US were 8.00 and 2.00 for HA-CDI and CA-CDI, respectively. The highest incidence in Europe was reported in Poland (HA-CDI: 6.18 per 10,000 patient days, CA-CDI: 1.4 per 10,000 patient days), the lowest from the UK, at 1.99 per 10,000 patient days and 0.56 per 10,000 patient days for HA-CDI and CA-CDI, respectively.

No clear trend for incidence over time emerged, with most countries reporting stable rates but some either a decrease or increase.

Rates of recurrent CDI varied based on geographical setting. The rate of recurrence was lower in community-associated disease compared to healthcare-associated disease.

Independent CDI risk factors identified common to both initial CDI and recurrent CDI included increasing age, antibiotic use, recent hospitalisation, and proton pump inhibitor (PPI) use. In addition, leukocyte count, length of hospital stays, and Charlson comorbidity index score featured as statistically significant risk factors for recurrent CDI, but these are not reported among the most common statistically significant risk factors for initial CDI.

**Conclusions:**

Despite considerable heterogeneity, evidence suggests substantial incidence of recurrent and primary CDI, even after considerable efforts in the last decade.

**Supplementary Information:**

The online version contains supplementary material available at 10.1186/s12879-021-06147-y.

## Background

*Clostridioides difficile* (formerly *Clostridium*), also known as *C. difficile,* is a Gram-positive anaerobic bacterium, which causes *Clostridioides difficile* infection (CDI). *The bacterial spores* are resistant to heat and numerous other disinfectants, which contributes to the spread of CDI in healthcare facilities. The infection starts with the symptoms of diarrhoea and can progress to life-threatening inflammation of the colon [1].

*Clostridioides difficile* has been recognised as a leading cause of healthcare-associated infections (HAIs)^1^ and a substantial threat to public health globally. The annual US economic burden is estimated at $796 million and the burden in European Union (EU) was estimated at €300 million per year [3, 4]. Aside from the substantial economic burden CDI imposes, the CDI incidence rate ranges from 1.1 to 631.8 per 100,000 population per year globally [5]. CDI is also associated with substantial morbidity and mortality worldwide, not only in some key risk groups (such as the elderly, hospitalised patients or those under antimicrobial treatment), but the general population as well [5, 6]. CDI is the leading cause of healthcare-associated infective diarrhoea and is increasingly being linked to community-acquired cases of colitis [7]. In addition to the considerable incidence of initial CDI episodes, recurrence is common and presents a substantial challenge. A recurrent episode is most commonly defined by international guidelines [8–11] as a re-occurrence of symptoms, within 8 weeks or less of a previous episode, provided the symptoms of the first occurrence have been resolved. Recent estimates put the rate of recurrence at around 15–35% of all CDI cases and data suggests second and subsequent recurrences are common among patients who experience a recurrent episode [12]. Recurrence leads to significant morbidity and healthcare costs that increase with each subsequent recurrence [12]. Risk factors for CDI and recurrent CDI (rCDI) include, among others, increasing age, which is a particularly important factor when exploring possible future trends in CDI incidence [5, 6].^2^ As many countries in the world face the challenges of an increasingly aging population, the elevated risk of CDI with increasing age means these countries will likely see their CDI incidence grow in the near future.

The use of antibiotics is another established risk factor for CDI, this is an important consideration given that their use also leads to increased antibiotic resistance [5, 6]. Despite the increasing challenge of antibiotic resistance, initial and recurrent CDI episodes are commonly recommended, by established international and national guidelines, to be treated with standard antibiotics such as vancomycin, while novel treatments such as faecal microbiota transplant (FMT) are reserved for multiple recurrent or more severe cases [8–11].

Given the current and expected future burden of CDI to health care systems, a systematic literature review (SLR) was conducted to identify epidemiology studies investigating CDI and rCDI. This study builds on the existing literature, notably Balsells et al. [5] who conducted a SLR and meta-analysis of initial CDI incidence, by assessing the incidence of recurrent cases (rCDI), trends in incidence over time, as well as risk factors for disease.

## Methods

The SLR was performed according to established methodologies and is presented here according to the Preferred Reporting Items for Systematic Reviews (PRISMA) guidelines [13].

For a detailed description of methods used to conduct the SLR, see Additional file.

### Scope of the search terms in the SLR

Search strategies were based on the combination of free text words, indexing terms (e.g. subject heading [EMTREE] or Medical Subject Headings [MESH] terms) and their relationship using Boolean terms (e.g. ‘and’, ‘or’, ‘not’). Searches were performed for the full CDI population (recurrent and initial disease). This ensured the search was kept broad to avoid excluding studies using differing terminology for recurrence.

### Search databases

The Ovid platform was used to conduct the literature searches [14]. Databases searched included Embase, MEDLINE and the Cochrane Library. Search terms are provided in Tables 2, 3 and 4 in Additional file.

Given CDI has become a healthcare focus in recent years, several public agencies and medical organisations have published studies and guidelines in this area, which may not be identified in a database search. Thus, additional hand searches of information published over the past 10 years in key websites were reviewed to ensure that all relevant evidence has been identified. These included national statistical offices or national agencies responsible for health reporting (e.g. Public Health England Database, Health Protection Scotland Database, CDAD-KISS Database in Germany), and international offices or national agencies responsible for health reporting (such as the European Centre for Disease Prevention and Control [ECDC] Epidemiology report [15]).

### Inclusion and exclusion criteria

The scope of search terms included disease terms for CDI (recurrent or initial) together with terms for epidemiology and disease-related findings. Included patients were adults with CDI (recurrent or initial). Studies from the following countries were considered for inclusion: EU-4 (France, Germany, Italy and Spain), UK, Poland, US, Canada, Australia, Japan and China. Study types included were observational studies and systematic literature reviews. Findings assessed in the review included patient demographics, CDI incidence (overall and by setting i.e. HA-CDI and CA-CDI), rate and number of recurrences and risk factors. Limits applied to the search were English language and data published between 2009 and 2019. Due to changes in diagnostics and definitions used, studies older than 10 years were not considered. Searches were carried out in January 2019.

### Quality assessment of included studies

The Newcastle-Ottawa Scale (NOS) was used to assess the quality of observational studies included in this review. The NOS is a validated tool to evaluate the risk-of-bias of non-randomised studies, including case-control and cohort studies [16, 17]. Domains assessed by the NOS comprise risk-of-bias in selection, comparability, and exposure/outcome. Studies can attain a grading of between zero and nine stars; zero to four stars for the selection domain, zero to two stars for comparability and zero to three stars for the exposure/outcome domain. If a study received zero or one star in the selection or exposure domain or zero stars in the comparability domain, the study was marked as “poor quality”. If a study fit the criteria of three or four stars in the selection domain, one or two stars in the comparability domain and two or three stars in the exposure/outcome domain, this study was marked as “good quality”. For all other permutations, studies were marked as “fair quality”.

## Results

### Study selection

The literature review identified 11,243 publications. Following deduplication, 8936 texts remained for title and abstract screening by two independent reviewers. Following title and abstract screening, and reconciliation between reviewers, 362 studies were included for full text review.

Of the 362 full-text papers screened, 12 did not concern the target population, 61 did not report a finding of interest, 96 were not of the eligible study design, while 28 duplications were removed, leaving 165 studies of interest. Additionally, the accompanying hand search identified a further 20 records deemed relevant for inclusion, leaving a total of 185 records included.

**Fig. 1:**
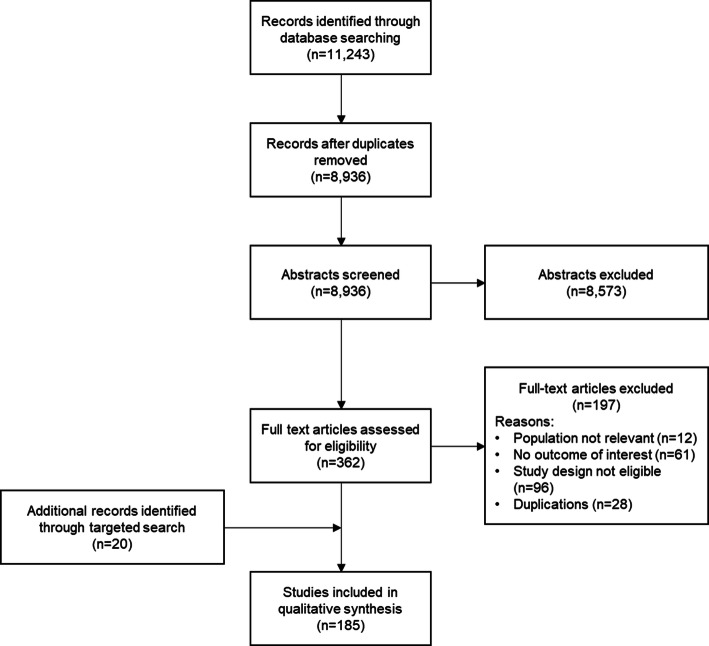
PRISMA diagram

The process of study selection is presented in the PRISMA flow diagram (Fig. 1). For a full list of included studies, see Table 6 in the Additional file.

### Quality assessment results

Out of 185 studies, 178 observational studies were quality assessed using the NOS, including 163 cohort studies and 16 case studies. Of the remaining seven studies, three were national surveillance databases, three were SLR and meta-analysis studies where the NOS is not suitable for use and the remaining study contained rates based on future projections. The NOS quality assessment score ranged from five to nine stars for the 178 assessed studies, with a mean score of seven stars (SD: 0.6). Of the 178 assessed studies, 92.1% (*n* = 164) were found to be “good quality” studies, 1.1% (*n* = 2) were marked as “fair quality” studies, and 6.7% (*n* = 12) were marked as “poor quality” studies. Among “poor quality” cohort studies (*n* = 11), commonly identified biases were found to be the lack of statement of follow-up of cohorts (11 studies [100%]), high risk of bias in ascertainment of exposure (10 studies [90.9%]) and assessment of outcome (5 studies [45.5%]). Only one case-control study was assessed as “poor quality” whose biases were found to be lack of statement of the non-response rate, inadequate case definition and high risk of bias in ascertainment of exposure. The quality assessment results for each included study is presented Table 6 in the Additional file.

### Study characteristics

Of the 185 included records, the majority focussed on the United States (83 studies [44.9%]). There were considerably fewer studies concerning other countries, in descending order by number of studies identified (with number and percentage of total, respectively); Italy (15 studies [8.1%]), Canada (15 studies [8.1%]), United Kingdom (12 studies [6.5%]), Australia (11 studies [5.9%]), Germany (10 studies [5.4%]), Spain (9 studies [4.9%]), China (7 studies [3.8%]), Japan (7 studies [3.8%]), France (6 studies [3.2%]) and Poland (4 studies [2.2%]). A further six studies (3.2%) had an international focus. Of the 185 included studies, 113 of them were categorised as large sample size studies (arbitrarily set to studies with > 1000 patients).

Among the screened studies, the most widely reported findings were initial CDI incidence (117 studies), recurrence rates (57 studies) and risk factors associated with CDI (50 studies). A number of reports also provided incidence rates stratified by onset (35 studies) or acquisition setting (49 studies).

The review also found that data pertaining to the point of treatment (i.e. the rates of hospital and community-treated CDI patients) was only reported in four studies [18–21]. The vast majority of studies were based on data collected from hospitals, this likely reflects evidence suggesting more CDI is healthcare-associated rather than community-associated; as well as the relative simplicity of studying disease in a hospital compared to a community setting. Studies were also inconsistent regarding definitions of disease, recurrence and acquisition and onset location, with a minority following ECDC definitions [22] and most not reporting on the definition used, which presents a limitation when interpreting results.

### Incidence of CDI and rCDI

#### Overall incidence rates

Incidence rates for primary and recurrent CDI were reported in 126 (68%) of included studies, with the following distribution by setting and acquisition; HA-CDI (33 studies, 18%), CA-CDI (20 studies, 11%), hospital-onset (HO-CDI) (21 studies, 11%) and community-onset (CO-CDI) (11 studies, 6%).

**Fig. 2:**
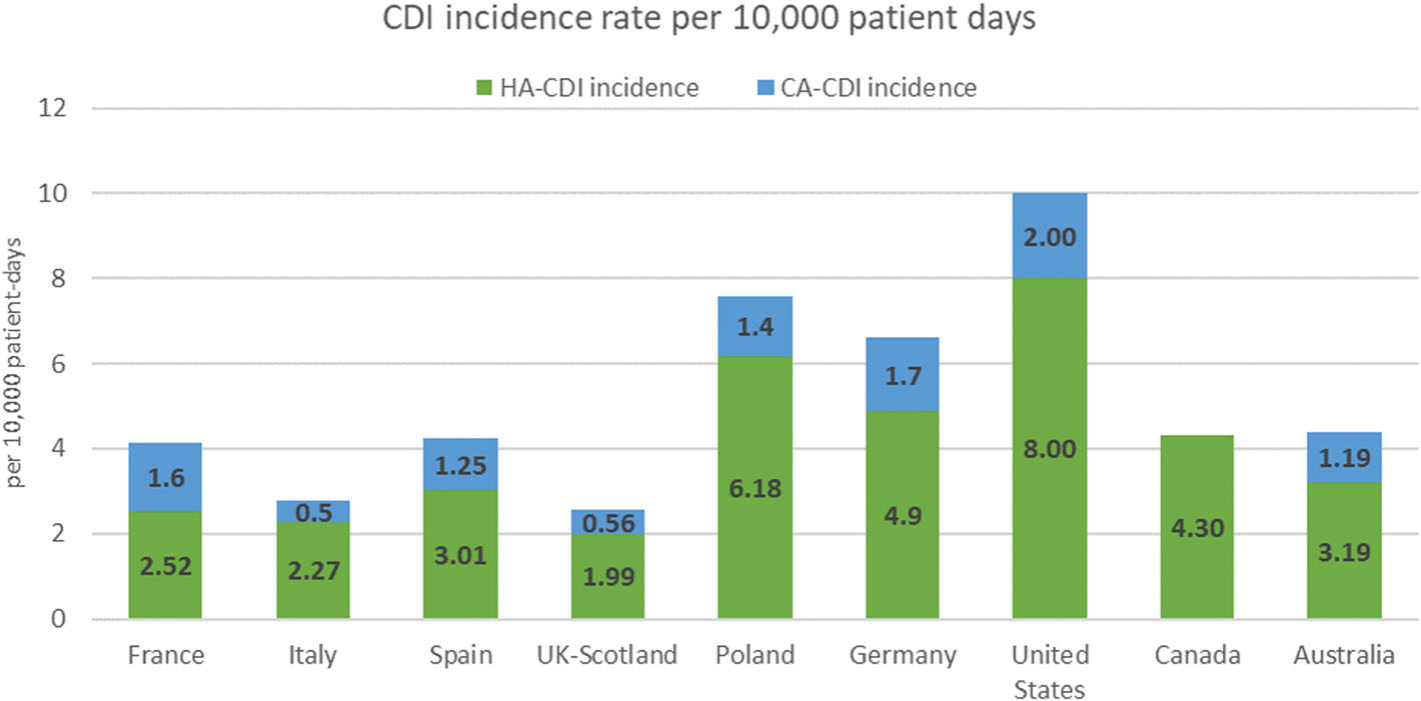
Overall CDI incidence per 10,000 patient days. *Sources:* ECDC 2018 [22], Katz et al. [23], Evans et al. [24], Gastmeier et al. [25], and Slimings et al. [26] *CA-CDI data in Australia paper was defined as non-HA-CDI (included CA-CDI and unknown CDI)

Of all the included studies across all territories, the median incidence per 10,000 patient days was 4.00 (0.30–74.4). For comparability, a key study, defined by sample size, reporting HA-CDI and CA-CDI incidence rates from each country of interest was identified (Fig. 2) [22–26]. Based on evidence captured in these key studies, CDI rates among countries of interest were highest in the US and Poland. Incidence rates per 10,000 patient days in the US were 8.00 and 2.00 for HA-CDI and CA-CDI, respectively. The highest rates in Europe were reported in Poland (HA-CDI: 6.18, CA-CDI: 1.4 per 10,000 patient days). The highest incidence rates among EU-5 countries were reported in Germany (HA-CDI: 4.9, CA-CDI: 1.7 per 10,000 patient days), while the lowest figures among these countries were from UK (pertaining to Scotland), at 1.99 and 0.56 per 10,000 patient days for HA-CDI and CA-CDI, respectively. The remaining EU5 countries demonstrated comparable rates (France: 2.52 and 1.6, Italy: 2.27 and 0.5, Spain: 3.01 and 1.25 per 10,000 patient days, for HA-CDI and CA-CDI, respectively). Data for Australia showed similar rates to those reported in Europe at 3.19 and 1.19 per 10,000 patient days for HA-CDI and CA-CDI, respectively. Finally, Canada reported a rate of 4.3 per 10,000 patient days for HA-CDI; a study reporting both HA-CDI and CA-CDI for Canada was not available (Fig. 2) [22–26]. The included sources did not specify patient days as hospital days, however, as data was collected from hospitals it is reasonable to assume the reported findings are per patient hospital days.

Incidence rates between countries showed considerable variation; the range between the lowest HA-CDI incidence reported (1.99 per 10,000 patient days in Scotland) and the highest (8.00 per 10,000 patient days in the US) was 6.01 per 10,000 patient days. The difference between lowest and highest reported CA-CDI rates (0.5 per 10,000 patient days in Italy and 2.00 in the US, respectively) was 1.50 per 10,000 patient days. These levels of difference may be attributed to variances in reporting practices and other factors such as the definitions used across territories and publications. Nonetheless, data shows CDI has a substantial incidence globally, with evidence suggesting higher rates for HA-CDI cases than for CA-CDI; on average, CA-CDI rates were reported as being 31.6% of HA-CDI rates.

##### Summary

Incidence rates per 10,000 patient days reported from all identified studies and only those studies considered to have a large sample size (> 1000 patients) are shown in Table 1. Medians and ranges are reported for each country as well as overall for all studies included. For countries with one paper identified, data from that study is reported in the tables.

**Table 1:** Overall incidence per 10,000 patient days from all identified studies and large size studies

Country	All identified studies			Large size studies		
	No.	Incidence		No.	Incidence	
		Median	Range		Median	Range
Australia	4	3.96	2.33–8.00	2	3.92	3.25–4.03
Canada	2	6.08	5.95–6.20	1	6.2	6.2
China	0	N/A	N/A	0	N/A	N/A
France	3	3.41	1.10–4.12	3	3.41	1.10–4.12
Germany	2	7.00	6.60–7.40	2	7	6.60–7.40
Italy	10	3.65	0.30–23.40	7	3.1	0.30–23.40
Japan	0	N/A	N/A	0	N/A	N/A
Poland	2	7.88	6.10–9.60	1	7.58	N/A
Spain	3	2.33	0.52–4.26	1	4.26	N/A
United Kingdom	5	7.10	2.32–74.40	4	4.82	2.32–19.80
United States	6	3.70	2.30–15.60	4	3.45	2.30–15.60
Overall	37	4.08	0.30–74.40	25	3.97	0.30–23.40

*Abbreviations*: *N/A* Not applicable, *No.* Number of key studies reporting outcome

#### Trends in incidence over time

**Fig. 3:**
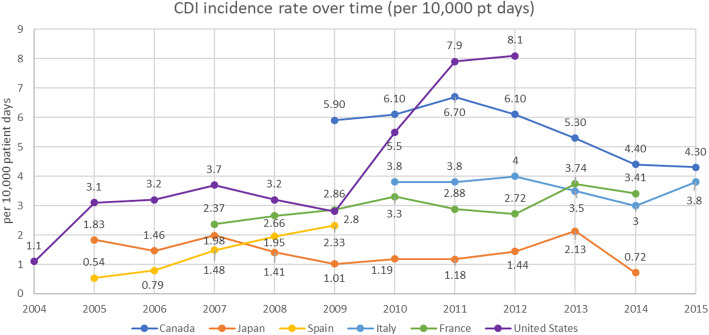
CDI incidence rate over time. Sources: Katz et al. [23], Roncarati et al. [30], Khanafer et al. [29], Guardiola et al. [27], Yoshikawa [31], and Kanamori [28]

Six studies (3.2% of all included studies) reported CDI incidence per 10,000 patient-days over time [23, 27–31] – Fig. 3 presents studies reporting CDI cases identified in hospitals over time. Overall, no clear trend emerged from the identified evidence, with some countries such as Canada reporting decreases over time, most reporting stability over time, but increases being observed in Spain until 2009 and in the US more recently from 2009 to 2012. Comparing the rates by country showed the US reported markedly higher rates (7.9 per 10,000 patient-days in 2011 to 8.1 per 10,000 patient-days in 2012) when compared to the rest of the countries, with the second highest rates reported in France (5.9 in 2009 to 4.3 in 2015).

This data is likely confounded by differences in reporting methods, diagnostic tests and differing definitions used throughout territories, as well as data relating to different time periods.

While incidence rates per 10,000 patient-days over time were not available for the UK and Germany, incidence trends from Public Health England [32] and German surveillance data for *C. diff*-associated diarrhoea, per 100,000 persons [33], were captured in the review. In England, while rates remained substantial, they were seen to taper between 2009 and 2016: NHS Trusts in England reported an incidence of 24 cases per 100,000 population (13,286 cases) in the 2017/18 financial year, which decreased by 76.1% in a decade (100.3 cases per 100,000 population, 55,498 cases in 2007/08). While Germany reported a comparably lower (53 per 100,000 population) rate in 2009, the figure surpassed the one reported in England, at 56 per 100,000 population in 2011, before slowly declining to 42 per 100,000 population in 2016.

The review found no discernible trend in incidence across countries in its scope; some showed stable incidence rates, while others showed decreasing or increasing rates over time.

#### Incidence of recurrent CDI (rCDI)

The most commonly used cut-off for a recurrent episode in the included studies were 8 weeks or less after a first episode, in line with international guidelines [8–11] as established earlier.

Data on incidence of rCDI was reported in 57 studies (30.8% of all included studies). The most common country reported was the US (49.1% of rCDI studies), with smaller numbers of studies available for European countries and Canada, and no studies reporting on Australia. Most studies reported overall recurrence rates without considering the number of events to have occurred.

Based on the reviewed literature, rCDI occurs in approximately 10–20% of incident CDI patients globally - the median recurrence rate from all included studies was 17.0%, with substantial variation across studies (range 0.0 to 64.0%). The highest rates were reported from Canada (median: 23.7%), Poland (median 21.7%), and the US (20.2%).

Data reporting the number of recurrent events was limited. Thirteen studies (7% of included studies) reported any finding relating to number of recurrences, with a variation in how many recurrences were considered, from one to four and subsequent. A 2016 Canadian study by Sheitoyan-Pesant [21], reported number of recurrences separately by the number of events occurring. Data pertained to four separate four-year periods between 1998 and 2013. During the total study period, 25% of initial CDI patients experienced a first recurrence, with around 38% of first recurrence patients then experiencing a second recurrence, 29% of patients with second recurrence experiencing a third recurrence, and 27% of third recurrence patients experiencing a fourth or additional recurrence.

Stratifying for the separate time periods revealed no clear trend for number of recurrences over time. Rates for one recurrence were reported as follows, by period: 12.8% (1998 to 2001), 30.2% (2002 to 2005), 19.7% (2006 to 2009) and 30.7% (2010 to 2013). Rates for two recurrences were 37.9% (1998 to 2001), 38.3% (2002 to 2005), 39.7% (2006 to 2009) and 37.3% (2010 to 2013). Rates for three recurrences were reported to be 30.0% (1998 to 2001), 29.2% (2002 to 2005), 37.5% (2006 to 2009) and 18.2% (2010 to 2013). Finally, four or more recurrences were reported in 27.8% (2002 to 2005), 25.0% (2006 to 2009) and 50.0% (2010 to 2013) of patients.

##### Recurrence rates by setting

Recurrence rates for HA-CDI ranged from 3.7 to 64.0%, while CA-CDI patients were reported to have between 3.1 and 28.0% recurrence rates. In all cases the rate of recurrence was lower in community-associated disease than healthcare-associated disease. This trend held across all countries with data and regardless of the definition of recurrence used.

##### Summary

A summary of findings for CDI overall recurrence rates reported from all studies and large sample size studies are shown in Table 2. Medians and ranges are reported for each country as well as overall for all studies included. For countries with one paper identified, data from that study is reported in the tables.

**Table 2:** Overall CDI recurrence rates from all studies and large size studies

Country	All identified studies			Large size studies		
	No.	Overall recurrence rate (%)		No.	Overall recurrence rate (%)	
		Median	Range		Median	Range
Australia	0	N/A	N/A	0	N/A	N/A
Canada	4	23.66%	10.40–36.05%	2	18.14%	10.40–28.84%
China	3	10.57%	3.06–13.33%	1	7.80%	3.06–8.00%
France	2	8.93%	6.10–11.76%	1	6.10%	N/A
Germany	4	17.14%	6.67–23.00%	1	18.15%	14.67–18.77%
Italy	4	14.60%	7.70–20.00%	1	15.26%	14.60–15.92%
Japan	4	5.40%	3.80–18.42%	2	11.61%	4.80–18.42%
Poland	1	21.70%	5.68–41.18%	1	21.70%	5.68–41.18%
Spain	5	12.71%	0.00–57.14%	1	57.14%	N/A
United Kingdom	2	19.32%	7.00–21.63%	1	21.63%	N/A
United States	28	20.22%	2.14–64.00%	16	17.45%	2.14–38.64%
Overall	57	17.00%	0.00–64.00%	27	17.34%	2.14–57.14%

*Abbreviation*: *N/A* Not applicable, *No.* Number of key studies reporting outcome

### Risk factors for CDI and rCDI

Of the 185 included studies, risk factors associated with CDI, either initial or recurrent infection, were reported by 50 (27.0%) studies. Most of the studies did not report whether the increase in risk was statistically significant. However, a number of key sources were captured that reported a statistically significant increase in CDI or rCDI risk.

Risk factors associated with initial CDI were reported by 31 (16.8%) studies. The most commonly identified, statistically significant, independent risk factors for initial infection across all studies were increasing age, antibiotic use, recent hospitalisation, being female, proton pump inhibitor use, having a feeding tube, being resident in a long-term care facility, malignant disease, having diabetes, having heart disease and steroid use (Table 3). When examining large studies only, the most commonly identified, statistically significant, independent risk factors for initial CDI also included Charlson comorbidity index score^3^ (Table 3).

**Table 3:** Most common statistically significant risk factors identified from all studies and large studies

Risk factor	Number of times reported as statistically significant			
	Initial CDI		rCDI	
	All studies	Large studies	All studies	Large studies
Age	19	16	11	6
Antibiotic use	18	13	6	3
Recent hospitalisation	11	8	3	3
Female	9	8	NA	NA
PPI use	7	7	5	2
Feeding tube	6	5	NA	NA
LTCF resident	5	4	NA	NA
Malignant disease	5	3	NA	2
Diabetes	4	4	4	2
Heart disease	4	3	NA	2
Steroid use	4	NA	NA	NA
Charlson comorbidity index	NA	3	4	2
Antibiotic risk index	NA	3	NA	NA
Ulcer	NA	3	NA	NA
Inflammatory bowel disease	NA	3	NA	NA
Leukocyte count	NA	NA	6	3
Hospital length of stay	NA	NA	4	3
Anti-acid medication use	NA	NA	3	NA
ICU admission	NA	NA	3	2

*Abbreviations*: *ICU* Intensive care unit, *LTCF* Long-term care facility, *N/A* Not applicable (due to this risk factor was not identified as one of the most common statistically significant factors for corresponding category), *PPI* Proton pump inhibitors

While odds ratios (OR) were not commonly reported, the review captured a study by Viale et al. [35], which included more than 10,000 hospitalised CDI patients in Italy. ORs for risk of CDI, reported in this study, by magnitude of increased risk, were 13.30 for previous CDI, 2.94 for antibiotic use, 2.88 for previous hospitalisations, 2.28 for female sex, 1.82 for PPI use, 1.82 for being a nursing home resident, 1.57 for at least 30 days for bed rest, 1.37 for every 10-year age increase and 1.28 for parenteral nutrition.

Risk factors for recurrent CDI were reported by 19 (10.3%) of the included studies. The most commonly identified, statistically significant, independent risk factors for recurrent infection across all studies are shown in Table 3. Of note, across all studies, leukocyte count, length of hospital stays, anti-acid medication usage and admission to an intensive care unit were found to be statistically significant risk factors for recurrent infection, which were not noted for initial infection (Table 3). When examining large studies only, the most commonly identified, statistically significant, independent risk factors for recurrent CDI also included having malignant disease and heart disease, which were also identified as independent risk factors for initial CDI (Table 3).

## Discussion

*Clostridioides difficile* is a leading cause of healthcare-associated infections and CDI presents an increasing healthcare problem, not just for affected patients but to healthcare systems and public health across the world. In addition to a substantial number of patients experiencing an initial episode, recurrences are prevalent and the limited options in the treatment arsenal present further challenges in the area.

Public health authorities worldwide have been enacting measures to monitor and combat CDI, such as mandatory surveillance and yearly case number targets [36]. In some instances, financial sanctions are imposed on institutions that exceed targets, as is the case in the UK, where Public Health England sets the yearly targets for care providers and each excess case incurs a financial sanction [36].

Antimicrobial resistance is the one of the greatest global health issues to tackle [37, 38]. The number, dosage, and exposure duration of prescribed antibiotics are associated with increasing risk of CDI [39]. Due to the prescribers’ education and habits, antibiotics tend to be over prescribed in current practice [40, 41]. Thus, antibiotic knowledge and responsible prescribing education plays an important role in minimising the risk of CDI [39, 42]. To avoid further worsening the already challenging antibiotic resistance seen globally, another area of focus is the implementation of antimicrobial stewardship programmes. These initiatives, such as guidance from the National Institute for Health and Care Excellence (NICE) in the UK [43], aim to limit antibiotic usage to where it is essential, to avoid antimicrobial resistance rendering current treatments ineffective. Currently, many standard therapies used in treating infections are broad spectrum antibiotics such as vancomycin, which has been a staple of CDI treatment [8–11]. Vancomycin is used across many indications due to its efficacy and low cost, which means patients with a range of illnesses may develop resistance to the treatment, presenting a challenge for its future use.

This literature review identified a number of data sources for the epidemiology of CDI. The evidence encompassed incidence of both primary infection and recurrence, as well as risk factors associated with infection. The findings of this review point to a substantial incidence of CDI globally, which is higher among patients in the healthcare setting, particularly for those who are hospitalised. Of countries of interest to this review, incidence rates were highest in the US and Poland, which could, at least in part, be explained by stronger reporting of CDI compared to some other countries. The differences may also be explained, by the different levels of use and access to broad-spectrum antibiotics, as well as their use as non-prescription medicines in some regions. Rates in Australia were comparable to those in the EU-5; while Canada showed slightly higher rates for HA-CDI. While incidence rates from single countries over time were scarce and no clear trends emerged, data available suggests that CDI is a persistent issue and there is no evidence to suggest it is diminishing.

Data showed that disease recurrence affects a considerable number of patients and is a global problem, numerous patients continue to have multiple recurrences, with each additional recurrence more likely to require even more costly hospital treatment. Numbers suggested that approximately a quarter of patients will have a recurrence, with more than a third of these patients experiencing a second episode. Notably, further recurrences were also commonly reported, with close to a third of patients at risk for third and fourth or subsequent episodes [21]. This finding underlines the need for effective treatments against recurrences to lower the rates at which patients experience another episode. Preventing recurrences is especially important given the high rate of subsequent recurrences, which means a considerable percentage of patients will experience multiple recurrences that could be avoided with effective prevention after a first episode.

Consistent with the findings of other recent reviews, certain patient groups, such as the elderly and those with prior antibiotic exposure, are more at risk of both initial and recurrent CDI [44–46]. Being female was reported as being one of the most common statistically significant risk factors for initial CDI but did not feature in the list of most common statistically significant risk factors for recurrent CDI. Interestingly, leukocyte count features as a common statistically significant risk factor for recurrent CDI but is not reported as a common risk factor for initial CDI.

These findings, taken together, underline that CDI is, and will likely remain for the foreseeable future, a disease with a large burden, for which there is a need for effective treatments, both for first episodes and recurrences. Effective measures against antimicrobial resistance, such as antibiotic stewardship programmes, are an important part of combating this infection as the currently available treatment options are limited and are largely made up of standard antibiotics.

Notable limitations of the study included the varying availability of data by territory (i.e. the large majority were from the US) and by finding (i.e. incidence rates and risk factors were reported relatively commonly, while other findings such as time trends and point of treatment were less frequent). Another limitation was the inconsistency among studies, in whether they reported definitions used and if so, what definitions were adhered to, which limits the generalisability of some of the comparisons that can be made from the data. Furthermore, incidence data showed large variations across countries, which may be attributed to different reporting practices or different diagnostic tests used, rather than raw incidence rate differences. Finally, this SLR was limited to English language studies that were conducted in the specified 11 countries; the authors acknowledge that further data may have been available in publications in other languages and other country settings.

## Conclusions

In conclusion, despite a marked heterogeneity in the identified evidence, the findings of the review point to a substantial incidence of CDI, with the majority of disease occurring in hospitalised patients. Incidence rates appeared to be highest in the Poland and US, though the limitations discussed above apply. The identified evidence suggests CDI is a disease with a substantial burden, and it shows an unmet need for antibiotic stewardship and new treatments with novel methods of action to combat new and emerging strains of disease, as well as antibiotic resistance.

## Supplementary Information

**Additional file 1.**

## Data Availability

The full dataset is available from the corresponding author upon request.

## Abbreviations

CA-CDI: Community-acquired CDI
CDC: Centres for Disease Control and Prevention
CDAD-KISS: *Clostridium difficile*-associated Diarrhoea Component of [German Nosocomial Infection Surveillance System] KISS
CDI: *Clostridioides difficile* infection
CO-CDI: Hospital-onset CDI
ECDC: European Centre for Disease Prevention and Control
EU: European Union
EU5: European Union Five
FMT: Faecal microbiota transplant
HA-CDI: Hospital-acquired CDI
HAI: Healthcare associated infection
HO-CDI: Community-onset CDI
NICE: National Institute for Health and Care Excellence
NOS: Newcastle-Ottawa Scale
PRISMA: Preferred Reporting Items for Systematic Reviews and Meta-Analyses
rCDI: Recurrent *Clostridioides difficile* infection
SLR: Systematic literature review
UK: United Kingdom
US: United States

## Footnotes

1 HAI is a broad term that encompasses all infections that occur while receiving health care, whether in a hospital or other care facility [2]. Specific categories of infections by setting are discussed in the “Recurrence rates by setting” section.

2 Evidence for risk factors by setting was not captured in the review. The possible differences in population characteristics between hospital and community settings may be a source of confounding for age as a risk factor for CDI.

3 Charlson comorbidity index score is one of the most widely used comorbidity weighted indices to predict the risk of mortality [34].

## References

[1] Smits WK, Lyras D, Lacy DB, Wilcox MH, Kuijper EJ (2016). Clostridium difficile infection. Nat Rev Dis Primers.

[2] Haque M, Sartelli M, McKimm J, Abu Bakar M (2018). Health care-associated infections - an overview. Infect Drug Resist.

[3] McGlone SM, Bailey RR, Zimmer SM, Popovich MJ, Tian Y, Ufberg P (2012). The economic burden of Clostridium difficile. Clin Microbiol Infect.

[4] Jones AM, Kuijper EJ, Wilcox MH (2013). Clostridium difficile: a European perspective. J Inf Secur.

[5] Balsells E, Shi T, Leese C, Lyell I, Burrows J, Wiuff C, Campbell H, Kyaw MH, Nair H (2019). Global burden of Clostridium difficile infections: a systematic review and meta-analysis. J Glob Health.

[6] Lessa FC, Gould CV, McDonald LC (2012). Current status of Clostridium difficile infection epidemiology. Clin Infect Dis.

[7] Freeman J, Bauer MP, Baines SD, Corver J, Fawley WN, Goorhuis B, Kuijper EJ, Wilcox MH (2010). The changing epidemiology of Clostridium difficile infections. Clin Microbiol Rev.

[8] Debast SB, Bauer MP, Kuijper EJ (2014). European Society of Clinical Microbiology and Infectious Diseases: update of the treatment guidance document for Clostridium difficile infection. Clin Microbiol Infect.

[9] McDonald LC, Gerding DN, Johnson S, Bakken JS, Carroll KC, Coffin SE (2018). Clinical practice guidelines for Clostridium difficile infection in adults and children: 2017 update by the Infectious Diseases Society of America (IDSA) and Society for Healthcare Epidemiology of America (SHEA). Clin Infect Dis.

[10] Sartelli M, Malangoni MA, Abu-Zidan FM, Griffiths EA, Di Bella S, McFarland LV (2015). WSES guidelines for management of Clostridium difficile infection in surgical patients. World J Emerg Surg.

[11] Surawicz CM, Brandt LJ, Binion DG, Ananthakrishnan AN, Curry SR, Gilligan PH, McFarland LV, Mellow M, Zuckerbraun BS (2013). Guidelines for diagnosis, treatment, and prevention of Clostridium difficile infections. Am J Gastroenterol.

[12] Singh T, Bedi P, Bumrah K, Singh J, Rai M, Seelam S (2019). Updates in treatment of recurrent Clostridium difficile infection. J Clin Med Res.

[13] Moher D, Liberati A, Tetzlaff J, Altman DG, Group P (2009). Preferred reporting items for systematic reviews and meta-analyses: the PRISMA statement. BMJ.

[14] Ovid Search Platform. 2020. Available from: https://ovidsp.ovid.com/. Accessed Jan 2019.

[15] European Centre for Disease Prevention and Control (2018). Surveillance and disease data for *Clostridium difficile* infections.

[16] Luchini C, Stubbs B, Solmi M, Veronese N (2017). Assessing the quality of studies in meta-analyses: advantages and limitations of the Newcastle Ottawa scale. World J Meta-Anal.

[17] Wells G, Shea B, O'Connell D, Peterson J, Welch V, Losos M (2019). Newcastle-Ottawa Scale (NOS) for assessing the quality of nonrandomised studies in meta-analyses.

[18] Huebner N-O, Dittmann K, Henck V, Wegner C, Kramer A, Action Group Infection P (2016). Epidemiology of multidrug resistant bacterial organisms and Clostridium difficile in German hospitals in 2014: results from a nationwide one-day point prevalence of 329 German hospitals. BMC Infect Dis.

[19] Jurke A, Lunemann M, Friedrich AW, Daniels-Haardt I (2012). Description of notifications of severe cases of Clostridium difficile associated diarrhaea in North Rhine-Westphalia. Clin Microbiol Infect.

[20] Penit A, Bemer P, Besson J, Cazet L, Bourigault C, Juvin ME, Fix MH, Bruley des Varannes S, Boutoille D, Batard E, Lepelletier D (2016). Community-acquired Clostridium difficile infections. Med Mal Infect.

[21] Sheitoyan-Pesant C, Abou Chakra CN, Pépin J, Marcil-Héguy A, Nault V, Valiquette L (2015). Clinical and healthcare burden of multiple recurrences of Clostridium difficile infection. Clin Infect Dis.

[22] European Centre for Disease Prevention and Control (2018). Healthcare-associated infections: *Clostridium difficile* infections. ECDC. Annual epidemiological report for 2016.

[23] Katz KC, Golding GR, Choi KB, Pelude L, Amaratunga KR, Taljaard M, Alexandre S, Collet JC, Davis I, du T, Evans GA, Frenette C, Gravel D, Hota S, Kibsey P, Langley JM, Lee BE, Lemieux C, Longtin Y, Mertz D, Mieusement LMD, Minion J, Moore DL, Mulvey MR, Richardson S, Science M, Simor AE, Stagg P, Suh KN, Taylor G, Wong A, Thampi N, Canadian Nosocomial Infection Surveillance Program (2018). The evolving epidemiology of Clostridium difficile infection in Canadian hospitals during a postepidemic period (2009-2015). CMAJ.

[24] Evans ME, Simbartl LA, Kralovic SM, Jain R, Roselle GA (2014). Clostridium difficile infections in veterans health administration acute care facilities. Infect Control Hosp Epidemiol.

[25] Gastmeier P, Weitzel-Kage D, Behnke M, Eckmanns T (2009). Surveillance of Clostridium difficile-associated diarrhoea with the German nosocomial infection surveillance system KISS (CDAD-KISS). Int J Antimicrob Agents.

[26] Slimings C, Armstrong P, Beckingham WD, Bull AL, Hall L, Kennedy KJ, Marquess J, McCann R, Menzies A, Mitchell BG, Richards MJ, Smollen PC, Tracey L, Wilkinson IJ, Wilson FL, Worth LJ, Riley TV (2014). Increasing incidence of Clostridium difficile infection, Australia, 2011-2012. Med J Aust.

[27] Guardiola J, Lobaton Ortega T, Rodriguez-Moranta F, Rodriguez Alonso L, Parra Cancino C, Duenas E (2012). Nosocomial *Clostridium difficile* infection (CDI) incidence in two Spanish referral hospitals. Secular trend during the last decade. J Crohns Colitis.

[28] Kanamori H, Weber DJ, Dibiase LM, Sickbert-Bennett EE, Brooks R, Teal L (2015). Longitudinal trends in all healthcare-associated infections through comprehensive hospital-wide surveillance and infection control measures over the past 12 years: substantial burden of healthcare-associated infections outside of intensive care units and “other” types of infection. Infect Control Hosp Epidemiol.

[29] Khanafer N, Oltra L, Hulin M, Dauwalder O, Vandenesch F, Vanhems P (2016). *Clostridium difficile* infection in a French university hospital Eight years of prospective surveillance study. Medicine (United States).

[30] Roncarati G, Dallolio L, Leoni E, Panico M, Zanni A, Farruggia P (2017). Surveillance of Clostridium difficile infections: results from a six-year retrospective study in nine hospitals of a north Italian local health authority. Int J Environ Res Public Health.

[31] Yoshikawa I, Kumei S, Watanabe T, Kume K, Harada M (2016). The incidence and trend of *Clostridium difficile* infection at a Japanese university hospital from 2005 to 2014. United Eur Gastroenterol J.

[32] Public Health England (2018). *Clostridium difficile* (*C. difficile*) infection: annual data.

[33] National Reference Centre (NRZ) (2017). Modul CDAD-KISS Referenzdaten..

[34] Charlson ME, Pompei P, Ales KL, MacKenzie CR (1987). A new method of classifying prognostic comorbidity in longitudinal studies: development and validation. J Chronic Dis.

[35] Viale P, Frasson S, Cipollini F, Menichetti F, Petrosillo N, Brunati S (2016). Epidemiology and outcome of Clostridium difficile infections in patients hospitalized in internal medicine: findings from the nationwide FADOI-PRACTICE study. BMC Infect Dis.

[36] NHS Improvement (2019). CDI objectives for NHS Organisations for 2019.

[37] World Health Organization. Ten global health issues to track in 2021. Geneva; 2020. Available from: https://www.who.int/news-room/spotlight/10-global-health-issues-to-track-in-2021

[38] Centers for Disease Control and Prevention. Antibiotic Resistance Threats in the United States, 2019. Atlanta: U.S. Department of Health and Human Services, CDC; 2019.

[39] Brown KA, Langford B, Schwartz KL, Diong C, Garber G, Daneman N. Antibiotic prescribing choices and their comparative C. difficile infection risks: a longitudinal case-cohort study. Clin Infect Dis. 2020;72(5):836–44. 10.1093/cid/ciaa124 PMC7935390 32069358

[40] Daneman N, Campitelli MA, Giannakeas V, Morris AM, Bell CM, Maxwell CJ, Jeffs L, Austin PC, Bronskill SE (2017). Influences on the start, selection and duration of treatment with antibiotics in long-term care facilities. CMAJ.

[41] Fernandez-Lazaro CI, Brown KA, Langford BJ, Daneman N, Garber G, Schwartz KL (2019). Late-career physicians prescribe longer courses of antibiotics. Clin Infect Dis.

[42] Di Gennaro F, Marotta C, Amicone M, Bavaro DF, Bernaudo F, Frisicale EM (2020). Italian young doctors’ knowledge, attitudes and practices on antibiotic use and resistance: a national cross-sectional survey. J Glob Antimicrob Resist.

[43] National Institute for Health and Care Excellence (2018). NICE impact: antimicrobial resistance.

[44] Song JH, Kim YS (2019). Recurrent Clostridium difficile infection: risk factors, treatment, and prevention. Gut Liver.

[45] Furuya-Kanamori L, Stone JC, Clark J, McKenzie SJ, Yakob L, Paterson DL (2015). Comorbidities, exposure to medications, and the risk of community-acquired Clostridium difficile infection: a systematic review and meta-analysis. Infect Control Hosp Epidemiol.

[46] Zilberberg MD, Shorr AF, Wang L, Baser O, Yu H (2016). Development and validation of a risk score for Clostridium difficile infection in Medicare beneficiaries: a population-based cohort study. J Am Geriatr Soc.

