# Supplementary material for: Burden of Clostridioides difficile infection (CDI) - a systematic review of the epidemiology of primary and recurrent CDI
Source: BMC Infect Dis. 2021 May 19;21:456. doi: 10.1186/s12879-021-06147-y (PMC8135979; doi:10.1186/s12879-021-06147-y)
Supplement: Supplementary file 1 — Additional file 1. [file 12879_2021_6147_MOESM1_ESM.docx]

Burden of CDI - A Systematic Literature Review of the Epidemiology of CDI and rCDI – Additional File

# Detailed search strategy

## Selection of references

### Article review

All publications included in the review following the search were extracted to an Excel document with the title, year of publication, authors and abstract provided for review.

Step 1: Abstract Review

All references/publications were reviewed based on their abstracts and titles against a set of pre-defined PICOS criteria. All abstracts in Step 1 were reviewed by a single reviewer. All papers included at the end of this stage were retained for Step 3. The reason for papers exclusion was provided. Papers for which there was uncertainty were retained for Step 2.

Step 2: Abstract Review when inclusion/exclusion uncertain

All publications where there was uncertainty or disagreement about inclusion were assessed by a second reviewer. Included publications were retained for Step 3; excluded publications were disregarded. The reason for each exclusion was provided.

Step 3: Full Text Review

Publications included after abstract review (from Step 1 and 2) were ordered for a full review of the text. The full texts of these publications were judged once more by the reviewer against the PICOS criteria.

The approach for resolving uncertainty regarding inclusion of papers was the same as for Step 2 of the abstract review process.

### Eligibility

During the structured literature review process, defined inclusion/exclusion criteria, in the form of PICOS (Population, Interventions, Comparators, Outcomes and Study design), were applied to select appropriate articles. The criteria are presented in the Table 1.

Table 1. Selection criteria

| **PICOS** | **Inclusion** | **Exclusion** |
| --- | --- | --- |
| Population | Patients over 18 years of age with CDI (or related CD toxins) | - Non-human - Children/paediatric populations |
| Intervention /Comparators | Any | None |
| Outcomes | - Incidence and prevalence of CDI - Expected changes over time - Patient/disease characteristics (e.g. hospital/community associated) - Risk factors for disease | Any studies not providing detail on the specific outcomes of interest |
| Study design | - Observational studies: - Cross-sectional studies, including surveys - Longitudinal (prospective/retrospective) cohort studies - Case-control studies - Systematic literature reviews | Any designs not listed in the inclusion criteria |
| Limits | - Countries: EU-5 (United Kingdom, France, Germany, Italy and Spain), Poland, US, Canada, Australia, Japan and China - Language: English - Years: 2009-2019 | - Countries not listed in inclusion criteria - Non-English language - Articles published prior to 2009 - Non-human focus |

### Search strings

Table 2. Search strings: Epidemiology - MEDLINE 1946-2019

| **#** | **Search Terms** | **Hits** |
| --- | --- | --- |
|  | **Disease Terms: CDI** | |
| 1 | Clostridium difficile/ | 8364 |
| 2 | Clostridium Infections/ | 7508 |
| 3 | (Clostridium adj3 difficile*). ab,ti,kw. | 13092 |
| 4 | ((c adj1 diff*) or CDI). ab,ti,kw. | 13872 |
| 5 | (TcdA or TcdB). ab,ti,kw. | 802 |
| 6 | or/1-5 | 23870 |
|  |  | |
| 7 | exp epidemiology/ | 25406 |
| 8 | exp incidence/ | 239118 |
| 9 | exp prevalence/ | 263489 |
| 10 | exp epidemiologic studies/ | 2263230 |
| 11 | exp risk factor/ | 754172 |
| 12 | (inciden$ or prevalen$ or epidemiolog$). ab,ti,kw. | 1659079 |
| 13 | exp demography/ | 1406839 |
| 14 | or/7-13 | 4433615 |
|  |  | |
| 15 | #6(Disease terms) AND #14(Epidemiology terms) | 7123 |
| 16 | historical article/ or letter/ or editorial/ or case reports/ | 3545228 |
| 17 | 15 not 16 | 6395 |
| **18** | **Limit 17 to (human and English language and yr="2009-2019")** | **3300** |

Table 3. Search strings: Epidemiology - Embase 1988 to 2019

| **#** | **Search Terms** | **Hits** |
| --- | --- | --- |
|  | **Disease Terms: CDI** | |
| 1 | Peptoclostridium difficile/ | 3966 |
| 2 | exp Clostridium Difficile Infection/ | 12672 |
| 3 | (Clostridium adj3 difficile*).ab,ti,kw. | 17488 |
| 4 | ((c adj1 diff*) or CDI).ab,ti,kw. | 18400 |
| 5 | exp Clostridium difficile toxin A/ | 1554 |
| 6 | exp Clostridium difficile toxin B/ | 1514 |
| 7 | (TcdA or TcdB).ab,ti,kw. | 1048 |
| 8 | or/1-7 | 30961 |
|  | **Epidemiology Terms** | |
| 9 | exp epidemiology/ | 2850241 |
| 10 | exp incidence/ | 402554 |
| 11 | exp prevalence/ | 650479 |
| 12 | exp risk factor/ | 916127 |
| 13 | (inciden$ or prevalen$ or epidemiolog$).ab,ti,kw. | 2122471 |
| 14 | exp demography/ | 182358 |
| 15 | or/9-14 | 4420173 |
|  | **Disease terms and Epidemiology terms with restrictions** | |
| 16 | #8(Disease terms) AND #15(Epidemiology terms) | 11532 |
| 17 | historical article/ or letter/ or editorial/ or case reports/ | 1448950 |
| 18 | 16 not 17 | 11004 |
| **19** | **Limit 18 to (human and english language and yr="2009-2019")** | **7911** |

Table 4. Search strings: Epidemiology - Cochrane Database of Systematic Reviews

| **#** | **Search Terms** | **Hits** |
| --- | --- | --- |
|  | **Disease Terms: CDI** | |
| 1 | MeSH descriptor Clostridium difficile explode all trees | 28 |
| 2 | MeSH descriptor Clostridium Infections explode all trees | 37 |
| 3 | (Clostridium difficile or difficile):ti,ab,kw | 1897 |
| 4 | (#1 OR #2 OR #3) | 1933 |
|  | **Epidemiology Terms** | |
| 5 | MeSH descriptor epidemiology explode all trees | 798 |
| 6 | MeSH descriptor incidence explode all trees | 2106 |
| 7 | MeSH descriptor prevalence explode all trees | 1440 |
| 8 | MeSH descriptor risk factor explode all trees | 1766 |
| 9 | (incidence or prevalence or epidemiology):ti,ab,kw | 168074 |
| 10 | (#5 OR #6 OR #7 OR #8 OR #9) | 170282 |
|  | **Disease terms and Epidemiology terms with restrictions** | |
| **16** | **(#4 AND #10)** | **314** |

## Data extraction

Data extraction was performed using a template designed in advance in an MS Excel spreadsheet, which was pilot-tested on two randomly selected included studies and refined for each search. Information on study design, selection criteria, study population/patient characteristics and outcomes were extracted. The data to be extracted from the included studies comprised, but was not limited to, the variables outlined in Table 5. Where relevant the units (e.g., years or months), type of statistic (e.g., mean or median), indication of data spread (e.g., standard deviation [SD], standard error [SE] or interquartile range [IQR]) and definition of outcome are reported

Table 5. Variables extracted across studies

| **Study characteristics** | **Patients characteristics** | **Outcomes data** |
| --- | --- | --- |
| - Publication details (Author, Year, Title, Journal/Proceeding) - Study objective - Study design - Study country - Study population base - Eligibility criteria - Outcome data source (and care setting) - Study follow-up duration | - Population - Patient subgroups - Gender - Age: mean/median (SD/SE/range/IQR) - Disease recurrence status - Duration of disease: mean (SD) - CDI acquisition setting (hospital/community) - Main diagnosis if not CDI | - Patient demographics - Incidence - Expected changes - Risk factors |

## List of included studies

Table 6. Complete list of included studies

| Author, year | Study design | Years included | CDI/population type | Population definition | Total persons/data points (N) | CDI patients/data points (n) | Risk of bias | NOS total score |
| --- | --- | --- | --- | --- | --- | --- | --- | --- |
| **International** | | | | | | | | |
| Balsells 2019 (1) | Meta-analysis | 2005-2015 | Healthcare-associated, Healthcare-associated and hospital-onset, community-associated | Varies by studies included, but ones without case definitions were excluded | NR | NR | NA | NA |
| Bauer 2011 (2) | Observational study | 2008 | Inpatient | HA, occurring in a hospital or nursing home after 48 h of admission or within 4 weeks after discharge from such a facility CA, patient had not been admitted to a HCF in the previous 12 weeks | NA | 509 | Poor | 6 |
| Borren 2017 (3) | Meta-analysis | 2000-2016 | Inpatient and Outpatient | Varies by studies included, but ones without case definitions were excluded | NA | NA | NA | NA |
| European Centre for Disease Prevention and Control 2018 (4) | Surveillance report | 2016 | Healthcare-associated, community-associated | Community-associated CDI (CA-CDI) is defined as a case of CDI with onset of symptoms: Outside of Healthcare Facility AND without discharge from a Healthcare Facility within the previous 12 weeks; OR On the day of admission to a Healthcare Facility or on the following day AND not resident in a Healthcare Facility within the previous 12 weeks.  Healthcare-associated CDI (HA-CDI) is defined as a case of CDI with onset of symptoms: On day three or later, following admission to a Healthcare Facility on day one; OR In the Community within 4 weeks of discharge from a Healthcare Facility (including the current hospital or a previous stay in any other Healthcare Facility). | NR | 7,711 | Good | 7 |
| Furuya-Kanamori 2015 (5) | Meta-analysis | 1994-2011 | Community-associated | Varies by studies included, but ones without case definitions were excluded | 56,776 | NR | NA | NA |
| Mukherjee 2017 (6) | Epidemiology forecast | 2016-2026 | Inpatient | Projected diagnosed events of hospitalized CDI | NA | 1,735,000 (predicted) | NA | NA |
| **Australia** | | | | | | | | |
| Australian Commission on Safety and Quality in Health Care 2018 (7) | Surveillance report | 2006-2005 | Inpatient | CDI cases identified in hospital | NR | 11,667 | Good | 7 |
| Clohessy 2014 (8) | Retrospective observational study | 2011 | Community onset, healthcare and community associated | HA, positive stool culture at >72 h after admission; OR as symptom onset in the community OR a positive sample within 72 h of admission, AND with hospital contact in the previous 4 weeks.  CO, symptom onset in the community OR a positive stool culture within 72 hours (h) of admission, AND with no hospital contact in the previous 12 weeks. Probable community-onset infection was a subgroup defined as a positive stool culture between 72 and 120 h after admission in a patient presenting with diarrhoea where no alternate diagnosis was made AND with no hospital contact in the previous 12 weeks. This subgroup was included in the community-onset group for analysis. | NR | 127 | Good | 8 |
| Collins 2017 (9) | Case-control study | 2013-2014 | Community associated | CA, a patient with no history of hospital admission within the past 12 weeks | 96 | 77 | Good | 7 |
| Ferguson 2011 (10) | Observational study | 2009 | Healthcare-associated, Healthcare-associated and hospital-onset, community-associated | NR | 123,574 | NR | Poor | 6 |
| Foster 2014 (11) | Prospective, observational study | 2011-2012 | Inpatient | HO, onset of diarrhoea > 48 h after admission to a hospital  CO, diarrhoea in the community or 48 h or less after admission to the hospital | 2,170 | 80 | Good | 7 |
| Mitchell 2011 (12) | Retrospective study | 2006-2010 | Inpatient | HA-HO, if the stool specimen collection date was > 48 h after admission to a healthcare facility. | NR | 357 | Good | 6 |
| Mitchell 2012 (13) | Retrospective study | 2010-2011 | Community onset | HA-CO: symptom onset occurred in the community or 48 h after admission to a healthcare facility, provided that the onset of symptoms was less than 4 weeks after the last discharge from a healthcare facility where the patient had had a length of stay of 48 h. | NR | 438 | Good | 8 |
| Mitchell 2014 (14) | Retrospective observational study | 2007-2010 | Inpatient | Hospitalised for more than 48 hours | 58,942 | 158 | Good | 7 |
| Slimings 2014 (15) | Prospective surveillance study | 2011-2012 | Healthcare and community associated, inpatient | HA, diagnosis > 48 h after admission to a hospital, or < 48 h after admission to a hospital but < 4 weeks after the last discharge from a hospital  CA, symptom onset in the community or < 48 h after admission to a hospital provided that symptom onset occurred > 12 weeks after last discharge from a hospital | NR | 12,683 | Good | 8 |
| Slimings 2015 (16) | Cohort study | 2011-2012 | Healthcare-associated | NR | 345,356 | 824 | Good | 7 |
| Worth 2016 (17) | Observational study | 2010-2014 | Healthcare and community associated | HA-HO, >48 h after admission HA-CO, onset within 48 h of HCF admission and within 4 weeks of discharge from an HCF | NR | 6,736 | Good | 7 |
| **Canada** | | | | | | | | |
| Allard 2011 (18) | Retrospective study | 2005-2006 | Community associated | CA, patient had not been hospitalized for > 72 h at the time of the positive test result (if hospitalized), was not experiencing a recurrence (within 8 weeks) and had not been in contact with the healthcare system in the previous 4 weeks. | NR | 2,297 | Good | 7 |
| Babey 2015 (19) | Retrospective, population-based study | 2012-2014 | Healthcare and community associated | HA, onset of symptoms and positive testing > 48 hours after admission  CA, no hospital admission or by onset of symptoms and positive testing within 48 h of hospital admission. | NR | 29 | Good | 7 |
| Beausejour 2010 (20) | Observational prospective cohort study | NR | Healthcare-associated | NR | NR | 86 | Good | 7 |
| Brown 2015 (21) | Retrospective study | 2010-2014 | Inpatient | Acute care hospital stay that exceeded 2 days | 34,298 | 255 | Good | 7 |
| Daneman 2015 (22) | Retrospective cohort study | 2011-2012 | Healthcare-associated | NR | 653,896 | 2,341 | Good | 7 |
| Gilca 2010 (23) | Retrospective and prospective ecological study | 1998-2006 | Inpatient | NR | NR | 40,190 | Good | 7 |
| Gravel 2009 (24) | Prospective surveillance study | 2004-2005 | Healthcare-associated | HA, symptoms occurred at least 72 h after hospital admission or if symptoms resulted in readmission of a patient who had been hospitalized within the 2 months before the symptom onset date and who was not a resident in a long-term care facility or nursing home. | NR | 1,430 | Good | 7 |
| Katz 2018 (25) | Prospective surveillance study | 2009-2013 | Healthcare-associated | HA, a positive laboratory confirmation of C. difficile infection and a compatible clinical syndrome developing 72 h or longer after admission, or < 72 h after admission if patients had been previously admitted to the admitting hospital and discharged within the previous 4 weeks. | NR | 17,202 | Good | 7 |
| Lambert 2009 (26) | Observational study | 2005-2006 | Healthcare and community associated | HA-HO, a toxin- positive specimen collected > 48 h after admission to a HCF and before discharge.  HA-CO, toxin-positive specimen collected while in the community or within 48 h after admission to a HCF, provided discharge from a HCF < 4 week prior. | NR | 1,006 | Good | 7 |
| Loo 2011 (27) | Prospective study | 2006-2007 | Healthcare-associated | Healthcare associated CDI cases | 4143 | 117 | Good | 6 |
| Martin 2018 (28) | Point-prevalence survey | 2010, 2012, 2016 | Inpatient | HA, Onset of symptoms ≥ 72 h after admission to the hospital, OR the patient had been hospitalized in the hospital, and last discharged less than 8 weeks prior to the date the C. difficile toxin was first positive. HA, (another facility): Onset of patient’s symptoms is < 72 h after admission to the hospital AND the patient was hospitalized in and discharged from another hospital or long-term care facility less than 8 weeks before the date of admission to the same facility. CA, Onset of symptoms is < 72 h after admission to the hospital AND patient had not been hospitalized or in a long-term care facility in the past 8 weeks. | 35,018 | 436 | Good | 7 |
| Sheitoyan-Pesant 2015 (29) | Observational study | 1998-2013 | Inpatient | CDI cases identified in hospital | NR | 1527 | Good | 7 |
| Simor 2013 (30) | National point prevalence survey | 2010 | Inpatient | HA, onset of symptoms ≥72 h after hospital admission or if the patient had been discharged from a hospital or along-term care facility within 8 weeks of the current hospital admission | 2,895 | 485 | Good | 7 |
| Taylor 2016 (31) | Prevalence Survey | 2002, 2009 | Healthcare-associated | Hospital acquired infections | 9953 | 115 | Good | 7 |
| Williams 2015 (32) | Point-prevalence survey | 2012 | Inpatient | Hospitalized in an acute care ward | 29,042 | 410 | Good | 7 |
| **China** | | | | | | | | |
| Galaydick 2015 (33) | Observational study | 2011-2012 | Inpatient | NR | 111 | 31 | Good | 8 |
| Ho 2017 (34) | Cross-sectional study | 2006-2014 | Healthcare and community associated | HA, samples obtained >48 h after admission or those who were hospitalized in a HCF within the previous 4 weeks CA, not been hospitalized in a HCF within the previous 12 weeks | NR | 15,753 | Good | 8 |
| Huang 2014 (35) | Prospective observational study - for CDI incidence; followed by retrospective case-control study - for risk factors | 2008-2009 | Inpatient | NR | 240 | 90 | Good | 7 |
| Huang 2016 (36) | Prospective study | 2008-2010 | Inpatient | NR | 470 | 93 | Good | 7 |
| Lihua 2015 (37) | Retrospective case-control study | 2010-2013 | Inpatient | The symptoms developed after 48 hours of admission or within 4 weeks after discharge from a healthcare facility. The patient had not been admitted to a healthcare facility in the previous 12 weeks | 202 | 52 | Good | 8 |
| Tang 2018 (38) | Prospective study | 2013-2014 | Inpatient | NR | 315 | 45 | Good | 7 |
| Xu 2016 (39) | retrospective case-control study | 2013-2015 | Inpatient | NR | NR | 81 | Good | 8 |
| **France** | | | | | | | | |
| Eckert 2013 (40) | Prospective, multicentric survey | 2009 | Healthcare and community associated | NR | NR | 1,316 | Poor | 6 |
| Khanafer 2011 (41) | Prospective surveillance study | 2006-2010 | Healthcare and community associated | HA, symptom onset occurs more than 48 h after admission to, or within 4 weeks of discharge from, a HCF. CA, symptom onset occurs within 48 h of admission and over 4 weeks following discharge from a HCF. | NR | 218 | Poor | 5 |
| Khanafer 2016 (42) | Prospective observational study | 2007-2014 | Inpatient | CA, if CDI signs presented in the absence of previous hospitalization within the past 12 weeks in outpatients or in inpatients within the first 48 h of admission.  HA, if diarrhoea started more than 2 days after hospital admission or if symptoms occurred within 4 weeks of hospital discharge. | 8,753 | 590 | Good | 7 |
| Khanafer 2017 (43) | Nested case-control study in a prospective, observational, cohort study | 2011-2014 | Inpatient | CA, symptoms presented in the absence of a previous hospitalization within the previous 12 weeks.  HA, if diarrhoea started more than two days after hospital admission or if symptoms occurred within 4 weeks of hospital discharge. | 945 | 233 | Good | 8 |
| Ogielska 2015 (44) | Retrospective observational study | 2008-2012 | Community associated, inpatient | CA, positive for *Clostridioides* toxin in the first 48 h following hospitalization (on the condition that the patient had not previously stayed overnight in a healthcare setting within the past 3 months). | NR | 136 | Good | 8 |
| Penit 2016 (45) | Observational study | 2013-2014 | Community associated | HA, onset of symptoms - on day three or later, following admission to a healthcare facility on day one, OR - in the community within 4 weeks of discharge from a HCF (including the current hospital or a previous stay in any other healthcare facility). CA, onset of symptoms: - outside of HCF AND without discharge from a HCF within the previous 12 weeks, OR - on the day of admission to a HCF or on the following day AND not resident in a HCF within the previous 12 weeks. | NR | 27 | Good | 7 |
| **Germany** | | | | | | | | |
| Abdel Samie 2013 (46) | Retrospective study | 2006-2009 | Hospital onset | Symptoms began in the community or during the first 48 hours after hospital admission with no pre-hospitalization documented in the last 12 weeks, ambulatory CDAD was assumed. | NR | 124 | Poor | 5 |
| Gastmeier 2009 (47) | Prospective study | 2007 | Inpatient | HA, if symptoms develop more than 48 h after admission to hospital, or if onset of symptoms occurred in the community within 4 weeks after discharge from hospital. HA other facility, symptoms developed <48 h after the admission to hospital, or onset of symptoms in the community occurred within 4 weeks after discharge from another healthcare facility. | 652,971 | 2,856 | Good | 6 |
| Huebner 2016 (48) | Point-prevalence survey | 2014 | Inpatient | NR | 73,983 | 506 | Good | 7 |
| Jurke 2012 (49) | Local health departments submitted epidemiology data | 2008-2010 | Inpatient | NR | NR | 196 | Good | 7 |
| Lubbert 2016 (50) | Retrospective database secondary data analysis | 2012 | Healthcare and community onset | NR | 1,461,268 | 1,223 | Good | 7 |
| Meyer 2012 (51) | Prospective observational study | 2010 | Healthcare-associated | NR | 1,536,031 | 7,897 | Good | 8 |
| National Reference Centre 2017 (52) | Surveillance report | 2007-2016 | Inpatient | CDI inpatients cases | 34,652,487 | 121,965 | NA | NA |
| Ott 2012 (53) | CDI cases assessed prospectively, clinical data for the cases retrieved retrospectively | 2007-2010 | Inpatient | HA, symptoms occurred >72 h after admission | NR | 761 | Good | 7 |
| Weis 2011 (54) | Retrospective study | 2009-2010 | Inpatient | NR | NR | NR | Good | 7 |
| Weiss 2009 (55) | Retrospective study | 2007 | Healthcare-associated | NR | NR | 15 | Fair | 6 |
| **Italy** | | | | | | | | |
| Alicino 2016 (56) | Retrospective study | 2010-2014 | Inpatient | HA, occurring >3 days after hospitalization, or within 28 days after discharge. | NR | 388 | Good | 7 |
| Bertizzolo 2013 (57) | Retrospective study | 2008-2009 | Inpatient | Admitted to internal medicine ward | 5,696 | 146 | Good | 7 |
| Carfagna 2014 (58) | Retrospective cohort study | 2012-2013 | Inpatient | NR | NR | 85 | Poor | 6 |
| Carfagna 2018 (59) | Observational study | 2017 | Inpatient | NR | NR | 116 | Poor | 5 |
| Del Prete 2017 (60) | Retrospective cohort study | 2015-2016 | Inpatient | NR | 366 | 31 | Good | 6 |
| Di Bella 2013 (61) | Retrospective cohort study | 2006-2011 | Inpatient | NR | 4,951 | 402 | Good | 7 |
| Falcone 2018 (62) | Observational study | 2014 | Healthcare and community associated | HA, positive specimen was not collected in an outpatient setting or >3 days after hospital admission and had documented overnight stay in a healthcare facility (i.e., hospital or nursing home) in the previous 12 weeks.   CA, positive specimen was collected in an outpatient setting or ≤3 days after hospital admission with no documented overnight stay in a healthcare facility (i.e., hospital or nursing home) in the previous 12 weeks. | NR | 717 | Good | 7 |
| Mellace 2013 (63) | Retrospective study | 2008-2009 | Inpatient | NR | 6,069 | 204 | Good | 7 |
| Morandi 2013 (64) | Observational study | 2011 | Healthcare and community onset | HA, CDI with date of lab testing >2 days from admission within 28 day from discharge  CA, CDI with date of lab testing within 2 days from admission and after 84 days from possible previous discharge, or none admission | NR | 980 | Good | 7 |
| Roncarati 2017 (65) | Retrospective study | 2010-2015 | Inpatient | CA, CDI signs occurred within 48 h from admission of patients who had not stayed in another hospital/HCF in the previous 3 months.  HA, diarrhoea started from 48 h after hospital admission to 4 weeks after hospital discharge or within 48 h from admission of patients discharged from another HCF within the previous 4 weeks. | NR | 942 | Good | 7 |
| Rosanna 2017 (66) | Retrospective surveillance study | 2005-2015 | Inpatient | NR | NR | 1,160 | Good | 7 |
| Sansone 2009 (67) | Retrospective study | 2007 | Healthcare-associated, healthcare onset, inpatients | HA-HO, acute onset of > 3 loose stools/day that persisted for at least 2 days. | 2,610 | 13 | Good | 7 |
| Ventrucci 2011 (68) | Survey | 2010 | Inpatient | NR | NR | 148 | Good | 7 |
| Viale 2016 (69) | Prospective observational study | 2013-2014 | Healthcare and community associated | Recent previous hospitalization or nursing home residents or onset of diarrhoea 3 days or more after admission to hospital | 10,780 | 103 | Good | 7 |
| Visconti 2017 (70) | Retrospective cohort study | 2013-2015 | Healthcare and community associated, inpatient | CO, in the patients without an history of hospital admission in the previous 30 days and if the onset of symptoms occurred within 48 h of admission to the hospital  HA-CO, if outpatients had an hospital admission in the previous 30 days CO long term facility (LTCF) associated, positive history for LTCF in the previous 30 days | 1,846 | 314 | Poor | 6 |
| **Japan** | | | | | | | | |
| Hikone 2015 (71) | Retrospective study | 2011-2013 | Healthcare-associated | HO, either 1) patients who had developed CDI hospitalized for more than 3 days or 2) patients who had developed CDI within 48 hours of admission with history of admission in HCF in previous 12 weeks.  CA, Patients who were diagnosed with CDI within 48 hours after admission with no history of hospitalization in the previous 12 weeks | 2,193 | 76 | Good | 6 |
| Honda 2014 (72) | Retrospective cohort study | 2010-2012 | Inpatient | HA-HO, symptom onset more than 48 h after admission to hospital. CO, symptom onset prior to admission or ≤3 days from admission.  HA-CO, onset within 4 weeks of discharge from hospital with last inpatient HCF exposure from the study hospital Indeterminate, onset 5-12 weeks since discharge from last inpatient HCF exposure CA, CDI onset >12 weeks from last inpatient HCF exposure | NR | 126 | Good | 7 |
| Mizui 2013 (73) | Observational study | 2010-2011 | Inpatient | NR | 2,716 | 29 | Good | 8 |
| Mori 2015 (74) | Retrospective, case-control study | 2010-2014 | Community associated | CA, patient presenting in the outpatients setting with a diagnosis of CDI with no history of hospital discharge in the 12 weeks prior to the visit. | NR | 26 | Good | 7 |
| Takahashi 2014 (75) | Multimethod investigation including a case–control study and cohort study | 2010-2011 | Inpatient | HA - Developed CDI within 48 h after hospital admittance. | 1,903 | 1,025 | Good | 7 |
| Yoshikawa 2016 (76) | Retrospective cohort study | 2005-2014 | Hospital-onset, healthcare-associated | HA-HO, symptom onset > 48 h after admission to hospital. | 126,396 | 307 | Good | 6 |
| Yoshino 2014 (77) | Retrospective cohort study | 2009-2012 | Inpatient | Admitted to internal medicine ward | 1,445 | 98 | Good | 7 |
| **Poland** | | | | | | | | |
| Czepiel 2015 (78) | Retrospective study | 2008-2014 | Inpatient | NR | NR | 1,009 | Good | 7 |
| Dulny 2013 (79) | Case-control study | 2008 | Inpatient | NR | 487 | 266 | Fair | 7 |
| Narodowy Instytut Zdrowia Publicznego - Państwowy Zakład Higieny 2018 (80) | Surveillance report | 2013-2017 | Inpatient | General population | NR | 40,523 | NA | NA |
| Pituch 2015 (81) | Hospital-based surveillance | 2011-2013 | Inpatient | NR | NR | NR | Good | 7 |
| **Spain** | | | | | | | | |
| Alcalá 2011 (82) | Questionnaire-based study | 2007 | Inpatient | NR | 30,723,117 | NR | Poor | 6 |
| Alvarez-Lerma 2014 (83) | Retrospective analysis | 2012 | Healthcare and community associated | NR | NR | 68 | Good | 7 |
| Esteban-Vasallo 2016 (84) | Population-based cross-sectional study | 2003-2014 | Inpatient | NR | NR | 13,526 | Good | 7 |
| Guardiola 2012 (85) | Retrospective surveillance study | 2000-2009 | Inpatient | NR | NR | 278 | Good | 7 |
| Hernandez 2013 (86) | Observational study | 2011-2012 | Healthcare and community onset | NR | NR | 58 | Good | 7 |
| Larrainzar-Coghen 2016 (87) | Prospective study | 2006-2013 | Inpatient | NR | NR | 502 | Good | 7 |
| Larrainzar-Coghen 2018 (88) | Prospective observational study | 2006-2015 | Inpatient | NR | NR | 724 | Good | 7 |
| Rodríguez-Pardo 2013 (89) | Prospective hospital-based surveillance | 2009 | Inpatient | NR | NR | 362 | Good | 7 |
| Salva 2014 (90) | Retrospective study | 2010-2011 | Inpatient | NR | 1936 | 7 | Good | 7 |
| **United Kingdom** | | | | | | | | |
| Banks 2016 (91) | Retrospective study | 2013-2014 | Community associated | NR | NR | 256 | Poor | 5 |
| Cottom 2016 (92) | Retrospective surveillance study | 2015-2016 | Healthcare and community associated | NR | NR | 137 | Good | 8 |
| Eyre 2012 (93) | Retrospective study | 2006-2010 | Healthcare and community onset | NR | NR | 1,678 | Good | 7 |
| Fellmeth 2010 (94) | Retrospective, descriptive epidemiology study | 2008-2009 | Community onset | NR | NR | 58 | Good | 7 |
| Health Protection Scotland 2018 (95) | Surveillance report | 2015-2018 | Healthcare and community associated | General population | NR | 6,489 | Good | 7 |
| King 2017 (96) | Cross-sectional retrospective study | 2012 | Inpatient | NR | 13,371,542 | NR | Good | 7 |
| Marwick 2013 (97) | Cohort study and case-control study | 2008-2009 | Community associated | NR | 79,039 | 137 | Good | 8 |
| Public Health England 2018 (98) | Surveillance report | 2007-2017 | Healthcare and community onset | General population | NR | 239,449 | NA | NA |
| Rana 2015 (99) | Observational Study | 2010-2013 | Healthcare-associated | NR | NR | NR | Good | 6 |
| Reddy 2010 (100) | Retrospective study | 2003-2007 | Inpatient | NR | 45,412 | 6,286 | Good | 7 |
| Virgincar 2011 (101) | Retrospective study | 2010 | Inpatient | NR | NR | 89 | Good | 7 |
| Wiuff 2011 (102) | Surveillance study | 2007-2009 | Inpatient | NR | NR | 1,613 | Poor | 5 |
| **United States** | | | | | | | | |
| Ajayeoba 2013 (103) | Retrospective cohort study | 2010-2011 | Inpatient | NR | 23,386 | 57 | Good | 7 |
| Argamany 2016 (104) | Retrospective cohort study | 2001-2010 | Inpatient | NR | NR | 1,676,903 | Good | 8 |
| Armbruster 2012 (105) | Retrospective study | 2005-2009 | Healthcare and community associated | NR | NR | 232 | Good | 7 |
| Barrett 2018 (106) | Observational study | 2011-2015 | Inpatient | Discharged hospitalization patients | NR | NR | Good | 7 |
| Bengualid 2011 (107) | Retrospective cohort study | 2006-2008 | Community associated | CA, non-nursing home or haemodialysis patient, who had no hospital admissions within the last 6 m, and who had positive C difficile toxin assays within the first 3 d of admission. | NR | 324 | Good | 7 |
| Benoit 2011 (108) | Retrospective, multicentre cohort study | 2007-2008 | Healthcare and community onset | NR | NR | 4,585 | Good | 7 |
| Bradley 2017 (109) | Retrospective cohort study | 2013-2014 | Healthcare and community associated | NR | NR | 502 | Good | 7 |
| Brown 2015 (110) | Retrospective cohort study | 2006-2012 | Healthcare-associated | NR | NR | 90 | Good | 7 |
| Brown 2016 (111) | Multilevel longitudinal nested case–control study | 2006-2012 | Healthcare onset | Acute care–onset, positive C difficile toxin test result 3 or more days after admission to an acute care facility. | 47,342 | 6,012 | Good | 7 |
| Brown 2017 (112) | Multilevel longitudinal case-cohort design study | 2006-2012 | Healthcare-associated | The outcome was the incidence of facility-onset laboratory-identified C. difficile infection (CDI), defined as a person with a positive C. difficile test without a positive test in the prior 8 weeks. | 223,781 | 35,754 | Good | 8 |
| Cadena 2010 (113) | Retrospective cohort study | 2003-2005 | Inpatient | NR | NR | 129 | Good | 7 |
| Campbell 2009 (114) | Retrospective study | 2006 | Healthcare onset | HO, laboratory diagnostic test, endoscopy, or biopsy result positive for C. difficile > 48 h after admission to a HCF | NR | 14,329 | Good | 7 |
| Chitnis 2013 (115) | Laboratory-based and population-based surveillance observational study | 2009-2011 | Community associated | NR | 1,624 | 984 | Good | 7 |
| Dean 2012 (116) | Retrospective cohort study | 2005-2011 | Healthcare and community associated | NR | NR | 7,372 | Good | 7 |
| Delate 2015 (117) | Retrospective cohort study | 2005-2011 | Healthcare and community associated, outpatient | NR | NR | 1,201 | Good | 8 |
| Dubberke 2009 (118) | Prospective cohort study | 2004-2005 | Healthcare and community onset | HO, c positive toxin assays > 48 h after hospital admission.  HA-CO, positive toxin assays ≤ 48 h after hospital admission, provided that diagnosis occurred within 60 d after the last discharge from one of the study wards and there was no other inpatient healthcare exposure from the time of discharge to readmission. | NR | 247 | Good | 7 |
| Dubberke 2009 (119) | Retrospective study | 2000-2006 | Community onset, healthcare-associated | NR | NR | 5,695 | Good | 7 |
| Dubberke 2010 (120) | Multicentre prospective study | 2000-2006 | Healthcare and community associated, healthcare and community onset | HO, patients with symptom onset >48 h after admission to the HCF  HA-CO, symptom onset in the community or ≤48 h after admission to an HCF, provided that symptom onset was less than 4 weeks after the last discharge from the study hospital and there were no exposures to other HCFs between the last discharge and most recent admission.  CA-CO, symptom onset in the community or ≤ 48 h after admission to an HCF, provided that symptom onset was > 12 weeks after the last discharge from an HCF | NR | 6,906 | Good | 7 |
| Dubberke 2011 (121) | Retrospective observational study | 2000-2006 | Inpatient | NR | NR | 10,832 | Good | 7 |
| Dubberke 2016 (122) | Retrospective cohort study | 2008-2009 | Healthcare and community onset | NR | 1,465,927 | 9,401 | Good | 7 |
| Dumyati 2012 (123) | Observational study | 2008 | Community associated | NR | NR | 366 | Good | 8 |
| Evans 2014 (124) | Retrospective study | 2010-2012 | Inpatient | A hospital-onset HCFA (HO-HCFA) case was defined as any nonduplicate/nonrecurrent positive LabID event collected more than 48 hours after admission, and a clinically confirmed HO-HCFA (CC-HO-HCFA) case was defined as an HO-HCFA case with diarrhoea or histopathologic or colonoscopic evidence of pseudomembranous colitis. CC-HOHCFA cases were collected to estimate the number of individuals who actually had an illness consistent with CDI, because a positive laboratory result can sometimes be obtained from the stool of an asymptomatic patient.  A community-onset healthcare facility–associated (CO-HCFA) case was defined as any nonduplicate/nonrecurrent CDI LabID event collected 24 hours or less before to 48 hours or less after admission to the acute care facility in cases in which the patient had been discharged from the same facility within the past 28 days. A community-onset not-healthcare-facility associated (CO-notHCFA) case was defined exactly the same as a CO-HCFA case with the exception that the patient had not been discharged from the same facility within the past 28 days.  A community-onset healthcare facility–associated (CO-HCFA) case was defined as any nonduplicate/nonrecurrent CDI LabID event collected 24 hours or less before to 48 hours or less after admission to the acute care facility in cases in which the patient had been discharged from the same facility within the past 28 days. A hospital-onset HCFA (HO-HCFA) case was defined as any nonduplicate/nonrecurrent positive LabID event collected more than 48 hours after admission, and a clinically confirmed HO-HCFA (CC-HO-HCFA) case was defined as an HO-HCFA case with diarrhoea or histopathologic or colonoscopic evidence of pseudomembranous colitis. CC-HOHCFA cases were collected to estimate the number of individuals who actually had an illness consistent with CDI, because a positive laboratory result can sometimes be obtained from the stool of an asymptomatic patient.  A community-onset not-healthcare-facility associated (CO-notHCFA) case was defined exactly the same as a CO-HCFA case with the exception that the patient had not been discharged from the same facility within the past 28 days. | NR | 9,642 | Good | 7 |
| Freedberg 2013 (125) | Observational study | 2009-2012 | Inpatient | NR | NR | 894 | Good | 8 |
| Friedman 2012 (126) | Longitudinal study | 2007-2010 | Healthcare-associated | NR | 4,633 | 1,145 | Good | 7 |
| Garg 2013 (127) | Retrospective cohort study | 2005-2010 | Hospital and community associated | The hospital-acquired CDAD was defined as CDAD acquired at least 3 days after admission. The LTCF population involved anyone from a long-term care facility, old-age home, assisted-living facility or chronic nursing home, who did not have overnight hospitalization in the past 12 weeks.  Community-acquired CDAD was defined as anyone coming from home, with no overnight stay in an inpatient healthcare facility in at least the 12 weeks prior to symptom onset. | NR | 311 | Good | 7 |
| Guerrero 2011 (128) | Retrospective cohort study | 2007-2008 | Healthcare associated, healthcare-onset | Healthcare-onset (onset of symptoms within 30 days of hospital discharge)  Healthcare-associated (episodes occurring more than 30 days after LTCF admission in patients with no diagnosis of CDI in the past 3 months) | NR | 105 | Good | 7 |
| Haran 2018 (129) | Retrospective cohort study | 2012-2014 | Healthcare and community associated | NR | NR | 863 | Good | 9 |
| Hebert 2013 (130) | Retrospective cohort study | 2006-2010 | Inpatient | NR | 1,389 | 829 | Good | 7 |
| Hudspeth 2019 (131) | Observational study | 2011-2014 | Community associated | NR | NR | 1,752 | Good | 7 |
| Hunter 2016 (132) | Retrospective cohort study | 2012 | Nursing home onset | Nursing homes included freestanding physical rehabilitation, skilled nursing, and inpatient hospice facilities | NR | 262 | Good | 7 |
| Ihimoyan 2010 (133) | Retrospective cohort study | 2003-2008 | Inpatient | NR | NR | 439 | Good | 7 |
| Jacob 2017 (134) | Retrospective matched cohort study | 2008-2015 | Healthcare-associated | Long-Term Acute-Care Hospital (LTACH)-onset CDI was defined as a positive C. difficile stool test occurring on day 4 or later of a patient’s hospitalization at the LTACH. This definition was based on the National Healthcare Safety Network (NHSN) definition for healthcare-facility–onset CDI. | NR | 130 | Good | 8 |
| Jarvis 2009 (135) | Point-prevalence survey | 2008 | Healthcare and community associated | Case patients were categorized by the setting in which C. difficile was likely acquired, to account for recent evidence that suggests that healthcare facility-associated CDAD may have its onset in the community up to 4 weeks after discharge (Definition provide in Ref 9 study in the article)  Community-associated CDAD was defined by symptom onset more than 12 weeks after the last discharge from a healthcare facility. | 110,550 | 1,443 | Good | 6 |
| Kanamori 2015 (136) | Retrospective observational study | 2001–2012 | Inpatient | ICU and non-ICU | 16,579 | NR | Good | 7 |
| Kang 2014 (137) | Observational study | 2005-2011 | Healthcare-associated | NR | 10,070 | 685 | Good | 7 |
| Karanika 2017 (138) | Retrospective cohort study | 2013-2015 | Healthcare-associated | NR | 1,761 | 213 | Good | 7 |
| Khanna 2012 (139) | Retrospective cohort study | 1991-2005 | Healthcare and community associated | Hospital acquired: Symptom onset > 48 hours from admission and hospitalized in the last 4 weeks.  Community acquired: Symptom onset <48 hours from admission and no hospitalization in last 12 weeks after the last discharge from a hospital Indeterminate cases were included as community-acquired in this study. Infection was defined as indeterminate if symptom onset occurred between 4 and 12 weeks from a hospital dismissal | 416 | 385 | Good | 7 |
| Khanna 2016 (140) | Retrospective cohort study | 2006-2013 | Healthcare and community associated | NR | NR | 679 | Good | 7 |
| Khanna 2016 (141) | Retrospective Study | 2005-2009 | Inpatient | NR | 162,000,000 | 1,260,000 | Good | 7 |
| Kim 2011 (142) | Retrospective cohort study | 2004-2010 | Healthcare-associated | A new episode of CDI in the long-term care facility was classified as either Hospital-associated CDI (HACDI), episode of new-onset diarrhoea caused by CDI occurring in the long-term care facility more than 4 days after the patient's long-term care facility admission and within 30 days after the patient's hospital discharge or Long-term care facility-associated CDI (LACDI), episode of new-onset diarrhoea caused by CDI occurring more than 30 days after admission to a long-term care facility in a patient who had no episodes of CDI within the prior 3 months. | NR | 162 | Good | 7 |
| King 2011 (143) | Retrospective, single-centre, medical record review | 2009 | Healthcare and community associated, inpatient | NR | 11,010 | 115 | Good | 7 |
| Kroner 2017 (144) | Retrospective cohort study | 2004-2013 | Inpatient | NR | NR | 1,021,454 | Good | 7 |
| Kuntz 2011 (145) | Retrospective, nested, case-control study | 2004-2007 | Healthcare and community associated | NR | 3,724 | 684 | Good | 7 |
| Kuntz 2011 (146) | Retrospective cohort study | 2005-2008 | Healthcare and community onset | NR | NR | 2,879 | Good | 7 |
| Kuntz 2012 (147) | Retrospective cohort study | 2005-2008 | Community onset | NR | 3,067 | 1,712 | Good | 7 |
| Kutty 2010 (148) | Retrospective study | 2005 | Community associated, inpatient | NR | NR | 212 | Good | 8 |
| Lessa 2014 (149) | Observational study | 2010 | Healthcare and community associated | NR | NR | 10,062 | Good | 7 |
| Lessa 2015 (150) | Retrospective study | 2011 | Healthcare and community associated | NR | NR | 15,461 | Good | 8 |
| Luo 2018 (151) | Retrospective cohort study | 2005-2014 | Healthcare onset | HO, secondary diagnosis of CDI and length of hospital stay ≥2 days. | 318,703,355 | 3,337,910 | Good | 7 |
| Ma 2017 (152) | Retrospective cohort study | 2001-2012 | Healthcare and community onset | NR | 38,911,718 | 45,341 | Good | 7 |
| McNabb-Baltar 2013 (153) | Population-based cohort study | 2006-2009 | Inpatient | NR | NR | 372,237 | Good | 7 |
| McNabb-Baltar 2013 (154) | Observational study | 1998-2010 | Healthcare and community associated | NR | NR | 3,100,641 | Good | 7 |
| Miller 2016 (155) | Retrospective cohort study | 2005-2011 | Healthcare-associated | NR | 10,329,988 | 26,086 | Good | 7 |
| Murphy 2012 (156) | Retrospective study | 2000-2007 | Healthcare and community associated, hospital-onset | Hospital-onset CDI (HO-CDI) cases defined by CDI POA=N (CDI Present on Admission code = No)  HA-CDI cases defined as the sum of HO-CDI and PD-CDI  Community-associated CDI (CA-CDI) cases defined by CDI POA=Y (CDI Present on Admission code = Yes) with no prior history of hospitalization in the previous 12 weeks. | 1,768,686 | 10,750 | Good | 7 |
| Naggie 2010 (157) | Surveillance study | 2005 | Healthcare and community associated | NR | NR | 108 | Good | 7 |
| Naik 2012 (158) | Retrospective cohort study | 2007-2010 | Healthcare and community associated | NR | NR | 666 | Good | 7 |
| Olsen 2016 (159) | Retrospective study | 2009 | Healthcare and community onset | NR | 48,320,235 | 423,805 | Good | 7 |
| Olson 2015 (160) | Observational, retrospective analysis | 2008-2014 | Inpatient | NR | NR | 123 | Good | 7 |
| Pakyz 2014 (161) | Case-control study | 2009 | Healthcare-associated | A case of healthcare-associated *Clostridioides* difficile infection (HA-CDI) was defined as a discharged patient with any ICD-9-CM code for CDI (008.45) who had also received drug treatment for CDI (e.g. metronidazole or oral vancomycin) for at least 3 days starting on or after day 3 of hospitalization, with a hospital stay ≤90 days. | 14,134 | 5,967 | Good | 7 |
| Pant 2015 (162) | Cohort study | 2006-2010 | Inpatient | NR | 491,406,018 | 462,160 | Good | 6 |
| Parasa 2017 (163) | Retrospective observational study | NR | Inpatient | NR | NR | 106,250 | Good | 7 |
| Pawar 2012 (164) | Population-based surveillance study | 2010 | Healthcare-associated | NR | 425 | 394 | Good | 7 |
| Pechal 2016 (165) | Retrospective cohort study | 2001-2010 | Inpatient | NR | NR | 2,279,004 | Good | 7 |
| Press 2016 (166) | Retrospective study | 2013 | Healthcare and community associated | If a patient tested positive for CDI after 3 days of admission the patient was determined to have hospital-acquired CDI  If a patient tested positive for CDI within 3 days of admission the patient was determined to have community acquired CDI | 40,990 | 189 | Good | 7 |
| Reeves 2016 (167) | Retrospective study | 2012-2014 | Healthcare and community onset | Long-term care facility–onset cases were defined as residents with nonduplicate, nonrecurrent LabID events collected >48 hours after admission  Community-onset cases were defined as residents with LabID events collected during the same interval as the admission prevalence, but they did not include recurrent cases. | 100,800 | 1,558 | Good | 7 |
| Reveles 2014 (168) | Retrospective study | 2001-2010 | Inpatient | NR | NR | 2,196,446 | Good | 8 |
| Reveles 2017 (169) | Retrospective cohort study | 2002-2014 | Healthcare and community associated | NR | NR | 30,326 | Good | 7 |
| Reveles 2018 (170) | Retrospective cohort study | 2003-2014 | Healthcare and community associated | NR | NR | 30,326 | Good | 7 |
| Saffouri 2017 (171) | Retrospective cohort study | 2005-2014 | Inpatient | NR | 162,000,000 | NR | Good | 7 |
| Shivashankar 2012 (172) | Retrospective cohort study | 2007-2010 | Outpatient | NR | NR | 520 | Good | 7 |
| Smith 2015 (173) | Retrospective, observational cohort study | 2006-2010 | Inpatient | NR | NR | 474,513 | Good | 7 |
| Troppy 2019 (174) | Surveillance study | 2016 | Healthcare and community onset | NR | NR | 7,728 | Good | 7 |
| Verlee 2012 (175) | Retrospective study | 2002-2008 | Inpatient | NR | NR | 68,686 | Good | 7 |
| Watson 2018 (176) | Retrospective cohort study | 2015-2016 | Healthcare onset | HO, positive stool test collected on hospital day >3 | 1237537 | 4587 | Good | 7 |
| Winkler 2010 (177) | Observational study | 2010 | Inpatient | NR | NR | 57 | Good | 7 |
| Young-Xu 2015 (178) | Retrospective cohort study | 2009-2013 | Inpatient | HCF-onset, HCF-associated CDI: CDIs with onset during a hospitalization or a stay in a long-term or skilled nursing facility  Community-onset, Community-associated CDI: CDIs with onset in the community (outpatient setting) among patients with no history of hospitalization or a stay in a long-term or skilled nursing facility in the 12 weeks prior to onset date  Community-onset, HCF-associated CDI: CDIs with onset in the community (outpatient setting) who had a history of hospitalization or a stay in a long-term or skilled nursing facility in the 4 weeks prior to onset date | 5,355,424 | 10,207 | Good | 7 |
| Yu 2016 (179) | Population-based retrospective cohort study for incidence of CDI, followed by case-control study for secondary objective of examining mortality | 2008-2010 | Healthcare-associated | NR | 32,807 | 941 | Good | 7 |
| Zarowitz 2015 (180) | Retrospective case-control study | 2009-2010 | Healthcare-associated | Non-nursing home-acquired (NNH-Acquired): all recorded diagnoses of CDI that first appeared in a New Admission MDS Assessment, Medicare Readmission/Return MDS Assessment, Medicare 5-Day Assessment, or Medicare 14-Day Assessment with prescription claims data were considered to be NNH-Acquired.  Nursing home-acquired (NH-Acquired): The first appearance of the diagnosis of CDI on any other type of MDS assessment was considered to be NH-Acquired. | NR | 2,048 | Poor | 6 |
| Ziakas 2016 (181) | Retrospective cohort study | 2011 | Healthcare-associated | NR | 1,806,900 | 41,565 | Good | 8 |
| Zilberberg 2011 (182) | Retrospective study | 2000-2008 | Inpatient | NR | NR | 340,352 | Good | 7 |
| Zilberberg 2011 (183) | Observational study | 2007-2008 | Healthcare and community associated | NR | 1,351,156 | 9,803 | Good | 7 |
| Zilberberg 2014 (184) | Retrospective cohort study | 2003-2009 | Inpatient | HA-HO, symptom onset > 48 hours after admission to an HCF  HA, CO, symptom onset in the community or 48 h or less after admission to an HCF, provided that symptom onset was < 4 weeks after the last discharge from an HCF.  CA, symptom onset in the community or 48 h or less after admission to an HCF, provided that symptom onset was > 12 weeks after the last discharge from an HCF. | NR | 4,200 | Good | 8 |
| Zilberberg 2017 (185) | Population-based cohort study | 2011 | Nursing home residents | Age ≥65, previous CDI episode in an acute care setting and discharged to an NH | NR | 14,472 | Good | 7 |

## PRISMA 2009 Checklist

| **Section/topic** | **#** | **Checklist item** | **Reported on page #** |
| --- | --- | --- | --- |
| **TITLE** | | |  |
| Title | 1 | Identify the report as a systematic review, meta-analysis, or both. | 1 |
| **ABSTRACT** | | |  |
| Structured summary | 2 | Provide a structured summary including, as applicable: background; objectives; data sources; study eligibility criteria, participants, and interventions; study appraisal and synthesis methods; results; limitations; conclusions and implications of key findings; systematic review registration number. | 2-3 |
| **INTRODUCTION** | | |  |
| Rationale | 3 | Describe the rationale for the review in the context of what is already known. | 3-5 |
| Objectives | 4 | Provide an explicit statement of questions being addressed with reference to participants, interventions, comparisons, outcomes, and study design (PICOS). | 5 |
| **METHODS** | | |  |
| Protocol and registration | 5 | Indicate if a review protocol exists, if and where it can be accessed (e.g., Web address), and, if available, provide registration information including registration number. | N/A |
| Eligibility criteria | 6 | Specify study characteristics (e.g., PICOS, length of follow-up) and report characteristics (e.g., years considered, language, publication status) used as criteria for eligibility, giving rationale. | 6; Additional file |
| Information sources | 7 | Describe all information sources (e.g., databases with dates of coverage, contact with study authors to identify additional studies) in the search and date last searched. | 5-6; Additional file |
| Search | 8 | Present full electronic search strategy for at least one database, including any limits used, such that it could be repeated. | Additional file |
| Study selection | 9 | State the process for selecting studies (i.e., screening, eligibility, included in systematic review, and, if applicable, included in the meta-analysis). | Additional file |
| Data collection process | 10 | Describe method of data extraction from reports (e.g., piloted forms, independently, in duplicate) and any processes for obtaining and confirming data from investigators. | Additional file |
| Data items | 11 | List and define all variables for which data were sought (e.g., PICOS, funding sources) and any assumptions and simplifications made. | Additional file |
| Risk of bias in individual studies | 12 | Describe methods used for assessing risk of bias of individual studies (including specification of whether this was done at the study or outcome level), and how this information is to be used in any data synthesis. | N/A |
| Summary measures | 13 | State the principal summary measures (e.g., risk ratio, difference in means). | N/A |
| Synthesis of results | 14 | Describe the methods of handling data and combining results of studies, if done, including measures of consistency (e.g., I^2^) for each meta-analysis. | N/A |

# References

1. Balsells E, Shi T, Leese C, Lyell I, Burrows J, Wiuff C, et al. Global burden of Clostridium difficile infections: a systematic review and meta-analysis. Journal of global health. 2019;9(1):010407.

2. Bauer MP, Notermans DW, Van Benthem BH, Brazier JS, Wilcox MH, Rupnik M, et al. Clostridium difficile infection in Europe: A hospital-based survey. The Lancet. 2011;377(9759):63-73.

3. Borren NZ, Ghadermarzi S, Hutfless S, Ananthakrishnan AN. The emergence of Clostridium difficile infection in Asia: A systematic review and meta-analysis of incidence and impact. PLoS ONE. 2017;12 (5) (no pagination)(e0176797).

4. European Centre for Disease Prevention and Control. Healthcare-associated infections: Clostridium difficile infections. In: ECDC. Annual epidemiological report for 2016. 2018.

5. Furuya-Kanamori L, Stone JC, Clark J, McKenzie SJ, Yakob L, Paterson DL, et al. Comorbidities, exposure to medications, and the risk of community-acquired Clostridium difficile infection: a systematic review and meta-analysis. infection control & hospital epidemiology. 2015;36(2):132-41.

6. Mukherjee D, Ayodele, L. Epidemiology -Clostridium Difficile Infection - Mature Markets Data. 2017.

7. Australian Commission on Safety and Quality in Health Care. Monitoring the national burden of Clostridium difficile. Sydney: ACSQHC; 2018.

8. Clohessy P, Merif J, Post JJ. Severity and frequency of community-onset Clostridium difficile infection on an Australian tertiary referral hospital campus. International Journal of Infectious Diseases. 2014;29:152-5.

9. Collins DA, Selvey LA, Celenza A, Riley TV. Community-associated Clostridium difficile infection in emergency department patients in Western Australia. Anaerobe. 2017;48:121-5.

10. Ferguson JK, Cheng AC, Gilbert GL, Gottlieb T, Korman T, McGregor A, et al. Clostridium difficile laboratory testing in Australia and New Zealand: national survey results and Australasian Society for Infectious Diseases recommendations for best practice. Pathology. 2011;43(5):482-7.

11. Foster NF, Collins DA, Ditchburn SL, Duncan CN, van Schalkwyk JW, Golledge CL, et al. Epidemiology of Clostridium difficile infection in two tertiary-care hospitals in Perth, Western Australia: A cross-sectional study. New Microbes and New Infections. 2014;2(3):64-71.

12. Mitchell B, Ware C, McGregor A, Brown S, Wells A. Clostridium difficile infection in Tasmanian public hospitals 2006 - 2010. Healthcare Infection. 2011;16(3):101-6.

13. Mitchell BG, Wilson F, McGregor A. An increase in community onset Clostridium difficile infection: A population-based study, Tasmania, Australia. Healthcare Infection. 2012;17(4):127-32.

14. Mitchell BG. Clostridium difficile infection: Incidence in an Australian setting. Asian Nursing Research. 2014;8(3):213-8.

15. Slimings C, Armstrong P, Beckingham WD, Bull AL, Hall L, Kennedy KJ, et al. Increasing incidence of Clostridium difficile infection, Australia, 2011-2012. Medical Journal of Australia. 2014;200(5):272-6.

16. Slimings C, Mahe C, Riley TV. The epidemiology of C. Difficile infection in Western Australia, 2011-2012. Journal of Microbiology, Immunology and Infection. 2015;48(2 SUPPL. 1):S113.

17. Worth LJ, Spelman T, Bull AL, Brett JA, Richards MJ. Epidemiology of Clostridium difficile infections in Australia: Enhanced surveillance to evaluate time trends and severity of illness in Victoria, 2010-2014. Journal of Hospital Infection. 2016;93(3):280-5.

18. Allard R, Dascal A, Camara B, Letourneau J, Valiquette L. Community-acquired Clostridium difficile-associated Diarrhea, Montreal, 2005-2006: Frequency estimates and their validity. Infection Control and Hospital Epidemiology. 2011;32(10):1032-4.

19. Babey K, Kelton S, Milne WK, Muileboom J, Voth B, Kelly L, et al. Clostridium difficile infection in rural Ontario: a retrospective multisite population-based study. Canadian journal of rural medicine : the official journal of the Society of Rural Physicians of Canada = Journal canadien de la medecine rurale : le journal officiel de la Societe de medecine rurale du Canada. 2015;20(4):117-20.

20. Beausejour Y, Pichette G, Su S, Frost E, Lavergne V. Factors associated with recurrence of clostridium difficile infection. American Journal of Gastroenterology. 2010;105(SUPPL. 1):S142-S3.

21. Brown K, Valenta K, Fisman D, Simor A, Daneman N. Hospital ward antibiotic prescribing and the risks of Clostridium difficile infection. JAMA Internal Medicine. 2015;175(4):626-33.

22. Daneman N, Guttmann A, Wang X, Ma X, Gibson D, Stukel TA. The association of hospital prevention processes and patient risk factors with the risk of Clostridium difficile infection: A population-based cohort study. BMJ Quality and Safety. 2015;24(7):435-43.

23. Gilca R, Hubert B, Fortin E, Gaulin C, Dionne M. Epidemiological Patterns and Hospital Characteristics Associated with Increased Incidence of Clostridium difficile Infection in Quebec, Canada, 1998-2006. Infection Control and Hospital Epidemiology. 2010;31(9):939-47.

24. Gravel D, Miller M, Simor A, Taylor G, Gardam M, McGeer A, et al. Health care-associated Clostridium difficile infection in adults admitted to acute care hospitals in Canada: A Canadian nosocomial infection surveillance program study. Clinical Infectious Diseases. 2009;48(5):568-76.

25. Katz KC, Golding GR, Choi KB, Pelude L, Amaratunga KR, Taljaard M, et al. The evolving epidemiology of Clostridium difficile infection in Canadian hospitals during a postepidemic period (2009-2015). Cmaj. 2018;190(25):E758-E65.

26. Lambert PJ, Dyck M, Thompson LH, Hammond GW. Population-based surveillance of Clostridium difficile infection in Manitoba, Canada, by using interim surveillance definitions. Infection Control and Hospital Epidemiology. 2009;30(10):945-51.

27. Loo VG, Bourgault A-M, Poirier L, Lamothe F, Michaud S, Turgeon N, et al. Host and pathogen factors for Clostridium difficile infection and colonization. New England Journal of Medicine. 2011;365(18):1693-703.

28. Martin P, Abou Chakra CN, Williams V, Bush K, Dyck M, Hirji Z, et al. Prevalence of antibiotic-resistant organisms in Canadian Hospitals. Comparison of point-prevalence survey results from 2010, 2012, and 2016. Infection Control and Hospital Epidemiology. 2018;40(1):53-9.

29. Sheitoyan-Pesant C, Abou Chakra CN, Pépin J, Marcil-Héguy A, Nault V, Valiquette L. Clinical and healthcare burden of multiple recurrences of Clostridium difficile infection. Clinical Infectious Diseases. 2015;62(5):574-80.

30. Simor AE, Williams V, McGeer A, Raboud J, Larios O, Weiss K, et al. Prevalence of colonization and infection with methicillin-resistant Staphylococcus aureus and vancomycin-resistant Enterococcus and of Clostridium difficile infection in Canadian hospitals. Infection Control and Hospital Epidemiology. 2013;34(7):687-93.

31. Taylor G, Gravel D, Matlow A, Embree J, LeSaux N, Johnston L, et al. Assessing the magnitude and trends in hospital acquired infections in Canadian hospitals through sequential point prevalence surveys. Antimicrob Resist Infect Control. 2016;5:19.

32. Williams V, Simor AE, Kiss A, McGeer A, Hirji Z, Larios OE, et al. Is the prevalence of antibiotic-resistant organisms changing in Canadian hospitals? Comparison of point-prevalence survey results in 2010 and 2012. Clinical Microbiology and Infection. 2015;21(6):553-9.

33. Galaydick J, Xu Y, Sun L, Landon E, Weber SG, Sun D, et al. Seek and you shall find: Prevalence of Clostridium difficile in Wuhan, China. American Journal of Infection Control. 2015;43(3):301-2.

34. Ho J, Dai RZW, Kwong TNY, Wang X, Zhang L, Ip M, et al. Disease burden of Clostridium difficile infections in adults, Hong Kong, China, 2006-2014. Emerging Infectious Diseases. 2017;23(10):1671-9.

35. Huang H, Wu S, Chen R, Xu S, Fang H, Weintraub A, et al. Risk factors of Clostridium difficile infections among patients in a university hospital in Shanghai, China. Anaerobe. 2014;30:65-9.

36. Huang Y, Li Y, Nie Y. Clinical characteristics of Clostridium difficile-associated diarrhea among patients in a tertiary care center in China. Pakistan Journal of Medical Sciences. 2016;32(3):736-41.

37. Lihua Z, Danfeng D, Cen J, Xuefeng W, Yibing P. Clinical characterization and risk factors of Clostridium difficile infection in elderly patients in a Chinese hospital. Journal of Infection in Developing Countries. 2015;9(4):381-7.

38. Tang C, Li Y, Liu C, Sun P, Huang X, Xia W, et al. Epidemiology and risk factors for Clostridium difficile-associated diarrhea in adult inpatients in a university hospital in China. American Journal of Infection Control. 2018;46(3):285-90.

39. Xu Q, Xu L, Feng H, Zhang Y, Zheng X, Tang L. Clinical risk factors for clostridium difficile infection in a Chinese tertiary care hospital. International Journal of Clinical and Experimental Medicine. 2016;9(2):4446-52.

40. Eckert C, Coignard B, Hebert M, Tarnaud C, Tessier C, Lemire A, et al. Clinical and microbiological features of Clostridium difficile infections in France: the ICD-RAISIN 2009 national survey. Medecine et maladies infectieuses. 2013;43(2):67-74.

41. Khanafer N, Benet T, Vanhems P. Community versus healthcare-associated Clostridium difficile infection: A surveillance-based study. Clinical Microbiology and Infection. 2011;17(SUPPL. 4):S98-S9.

42. Khanafer N, Oltra L, Hulin M, Dauwalder O, Vandenesch F, Vanhems P. Clostridium difficile infection in a French university hospital Eight years of prospective surveillance study. Medicine (United States). 2016;95(23):e3874.

43. Khanafer N, Vanhems P, Barbut F, Luxemburger C. Factors associated with Clostridium difficile infection: A nested case-control study in a three year prospective cohort. Anaerobe. 2017;44:117-23.

44. Ogielska M, Lanotte P, Le Brun C, Valentin AS, Garot D, Tellier AC, et al. Emergence of community-acquired Clostridium difficile infection: The experience of a French hospital and review of the literature. International Journal of Infectious Diseases. 2015;37:36-41.

45. Penit A, Bemer P, Besson J, Cazet L, Bourigault C, Juvin ME, et al. Community-acquired Clostridium difficile infections. Medecine et Maladies Infectieuses. 2016;46(3):131-9.

46. Abdel Samie A, Traub M, Bachmann K, Kopischke K, Theilmann L. Risk Factors for Recurrence of Clostridium Difficile-Associated Diarrhoea. Hepato-Gastroenterology. 2013;60(126):1351-4.

47. Gastmeier P, Weitzel-Kage D, Behnke M, Eckmanns T. Surveillance of Clostridium difficile-associated diarrhoea with the German nosocomial infection surveillance system KISS (CDAD-KISS). International journal of antimicrobial agents. 2009;33 Suppl 1:S19-23.

48. Huebner NO, Dittmann K, Henck V, Wegner C, Kramer A. Epidemiology of multidrug resistant bacterial organisms and Clostridium difficile in German hospitals in 2014: Results from a nationwide one-day point prevalence of 329 German hospitals. BMC Infectious Diseases. 2016;16(1):467.

49. Jurke A, Lunemann M, Friedrich AW, Daniels-Haardt I. Description of notifications of severe cases of Clostridium difficile associated diarrhaea in North Rhine-Westphalia. Clinical Microbiology and Infection. 2012;18(SUPPL. 3):653.

50. Lubbert C, Zimmermann L, Borchert J, Horner B, Mutters R, Rodloff AC. Epidemiology and Recurrence Rates of Clostridium difficile Infections in Germany: A Secondary Data Analysis. Infectious Diseases and Therapy. 2016;5(4):545-54.

51. Meyer E, Gastmeier P, Weizel-Kage D, Schwab F. Associations between nosocomial meticillin-resistant Staphylococcus aureus and nosocomial Clostridium difficile-associated diarrhoea in 89 German hospitals. Journal of Hospital Infection. 2012;82(3):181-6.

52. National Reference Centre (NRZ). Modul CDAD-KISS Referenzdaten. 2017.

53. Ott E, Valentin S, Schwab F, Chaberny I. Severe versus mild clostridium difficile infections: What are the differences? International Journal of Medical Microbiology. 2012;302(SUPPL.1):137.

54. Weis B, Friedrich A, Weitzel-Kage D, Eckmanns T. Still increasing incidence of Clostridium difficile-infections in Germany-need for action. International Journal of Medical Microbiology. 2011;301(SUPPL. 1):31.

55. Weiss B, Kleinkauf N, Eckmanns T, An Der Heiden M, Neumann M, Michels H, et al. Risk factors related to a hospital-associated cluster of Clostridium difficile PCR ribotype 027 infections in Germany during 2007. Infection Control and Hospital Epidemiology. 2009;30(3):282-4.

56. Alicino C, Giacobbe DR, Durando P, Bellina D, Di Bella AM, Paganino C, et al. Increasing incidence of Clostridium difficile infections: Results from a 5-year retrospective study in a large teaching hospital in the Italian region with the oldest population. Epidemiology and Infection. 2016;144(12):2517-26.

57. Bertizzolo L, Domeniconi G, Fabio G, Jacchetti G, Serafino S, Formica S, et al. Analysis of nosocomial acquired Clostridium difficile infection in an Italian research and teaching hospital. Annali di igiene : medicina preventiva e di comunita. 2013;25(2):119-24.

58. Carfagna P, Caccese R, D'Ambrosio M, Bruno E, Placanica P, Moriconi L, et al. Incidence and mortality associated with Clostridium difficile infection at a tertiary care hospital in Rome. Italian Journal of Medicine. 2014;8(SUPPL. 2):23.

59. Carfagna P, Tarasi A, Diamanti M, Placanica P, Iannone ME, Bruno E, et al. Clostridium colitis: What's new and unresolved issues. Italian Journal of Medicine. 2018;12(2 Supplement 1):37.

60. Del Prete R, Ronga L, Addati G, Magrone R, Miragliotta G. Prevalence of Clostridium difficile and ribotype 027 infection in patients with nosocomial diarrhoea in Southern Italy. The new microbiologica. 2017;40(4):264-8.

61. Di Bella S, Musso M, Cataldo MA, Meledandri M, Bordi E, Capozzi D, et al. Clostridium difficile infection in Italian urban hospitals: Data from 2006 through 2011. BMC Infectious Diseases. 2013;13(1):146.

62. Falcone M, Tiseo G, Iraci F, Raponi G, Goldoni P, Delle Rose D, et al. Risk factors for recurrence in patients with Clostridium difficile infection due to 027 and non-027 ribotypes. Clinical Microbiology and Infection. 2018;25(4):474-80.

63. Mellace L, Consonni D, Jacchetti G, Del Medico M, Colombo R, Velati M, et al. Epidemiology of Clostridium difficile-associated disease in internal medicine wards in northern Italy. Internal and emergency medicine. 2013;8(8):717-23.

64. Morandi M, Buttazzi R, Marchi M, Morsillo F, Gagliotti C, Moro ML. Incidence estimate of Clostridium difficile infection in Emilia-Romagna Region by linkage of administrative and laboratory data. Antimicrobial Resistance and Infection Control. 2013;2(SUPPL. 1):33.

65. Roncarati G, Dallolio L, Leoni E, Panico M, Zanni A, Farruggia P. Surveillance of Clostridium difficile infections: Results from a six-year retrospective study in nine hospitals of a north Italian local health authority. International Journal of Environmental Research and Public Health. 2017;14(1):61.

66. Rosanna L, Mirella B, Maria L, Peter S, Elisabetta P, Richard A. Surveillance of clostridium difficile infections in a Northern Italian Hospital, 2005-2015: Trends and control measures. Antimicrobial Resistance and Infection Control. 2017;6(Supplement 3):52.

67. Sansone S, Aschbacher R, Staffler M, Bombonato M, Girardi F, Larcher C, et al. Nosocomial diarrhoea in adult medical patients: The role of Clostridium difficile in a North Italian acute care teaching hospital. Journal of Preventive Medicine and Hygiene. 2009;50(2):117-20.

68. Ventrucci M, Farruggia P, Pozzato P, Panico M, Descovich C. Clostridium difficile infection survey in NHS Hospitals of Bologna. Italian Journal of Medicine. 2011;5(2 SUPPL. 1):113.

69. Viale P, Frasson S, Cipollini F, Menichetti F, Petrosillo N, Brunati S, et al. Epidemiology and outcome of Clostridium difficile infections in patients hospitalized in Internal Medicine: Findings from the nationwide FADOI-PRACTICE study. BMC Infectious Diseases. 2016;16(1):656.

70. Visconti V, Brunetti G, Cuomo MR, Giordano A, Raponi G. Nosocomial-acquired and community-onset Clostridium difficile infection at an academic hospital in Italy: Epidemiology, recurrences and toxin genes distribution. Journal of Infection and Chemotherapy. 2017;23(11):763-8.

71. Hikone M, Ainoda Y, Tago S, Fujita T, Hirai Y, Takeuchi K, et al. Risk factors for recurrent hospital-acquired Clostridium difficile infection in a Japanese university hospital. Clinical and Experimental Gastroenterology. 2015;8:191-6.

72. Honda H, Yamazaki A, Sato Y, Dubberke ER. Incidence and mortality associated with Clostridium difficile infection at a Japanese tertiary care center. Anaerobe. 2014;25:5-10.

73. Mizui T, Teramachi H, Tachi T, Tamura K, Shiga H, Komada N, et al. Risk factors for Clostridium difficile-associated diarrhea and the effectiveness of prophylactic probiotic therapy. Pharmazie. 2013;68(8):706-10.

74. Mori N, Aoki Y. Clinical characteristics and risk factors for community-acquired Clostridium difficile infection: A retrospective, case-control study in a tertiary care hospital in Japan. Journal of Infection and Chemotherapy. 2015;21(12):864-7.

75. Takahashi M, Mori N, Bito S. Multi-institution case-control and cohort study of risk factors for the development and mortality of Clostridium difficile infections in Japan. BMJ open. 2014;4(9):e005665.

76. Yoshikawa I, Kumei S, Watanabe T, Kume K, Harada M. The incidence and trend of clostridium difficile infection at a Japanese university hospital from 2005 to 2014. United European Gastroenterology Journal. 2016;4(5 Supplement 1):A650-A1.

77. Yoshino Y, Seo K, Koga I, Kitazawa T, Ota Y. Types of antibiotics and the risk factor for Clostridium difficile infection: Japanese study. International Journal of Infectious Diseases. 2014;21(SUPPL. 1):354.

78. Czepiel J, Kedzierska J, Biesiada G, Birczynska M, Perucki W, Nowak P, et al. Epidemiology of Clostridium difficile infection: Results of a hospital-based study in Krakow, Poland. Epidemiology and Infection. 2015;143(15):3235-43.

79. Dulny G, Zalewska M, Mlynarczyk G. An analysis of risk factors of Clostridiumdifficile infection in patients hospitalized in the teaching hospital in 2008. Przeglad epidemiologiczny. 2013;67(3):445-551.

80. Narodowy Instytut Zdrowia Publicznego - Państwowy Zakład Higieny. CHOROBY ZAKAŹNE I ZATRUCIA W POLSCE W 2017 ROKU podstawowe tablice robocze – wstępne dane.; 2018.

81. Pituch H, Obuch-Woszczatynski P, Lachowicz D, Wultanska D, Karpinski P, Mlynarczyk G, et al. Hospital-based clostridium difficile infection surveillance reveals high proportions of PCR ribotypes 027 and 176 in different areas of Poland, 2011 to 2013. Eurosurveillance. 2015;20(38):30025.

82. Alcala L, Marin M, Martin A, Sanchez-Somolinos M, Catalan P, Pelaez MT, et al. Laboratory diagnosis of Clostridium difficile infection in Spain: A population-based survey. Journal of Hospital Infection. 2011;79(1):13-7.

83. Alvarez-Lerma F, Palomar M, Villasboa A, Amador J, Almirall J, Posada MP, et al. Epidemiological study of Clostridium difficile infection in critical patients admitted to the Intensive Care Unit. Med Intensiva. 2014;38(9):558-66.

84. Esteban-Vasallo MD, Naval Pellicer S, Dominguez-Berjon MF, Cantero Caballero M, Asensio A, Saravia G, et al. Age and gender differences in Clostridium difficile-related hospitalization trends in Madrid (Spain) over a 12-year period. European Journal of Clinical Microbiology and Infectious Diseases. 2016;35(6):1037-44.

85. Guardiola J, Lobaton Ortega T, Rodriguez-Moranta F, Rodriguez Alonso L, Parra Cancino C, Duenas E. Nosocomial Clostridium difficile infection (CDI) incidence in two Spanish referral hospitals. Secular trend during the last decade. Journal of Crohn's and Colitis. 2012;6(SUPPL. 1):S176.

86. Hernandez M, Ramos M, Lecuona M. Clostridium difficile infection in Tenerife Canary Island, Spain. Antimicrobial Resistance and Infection Control Conference: 2nd International Conference on Prevention and Infection Control, ICPIC. 2013;2(SUPPL. 1):032.

87. Larrainzar-Coghen T, Rodriguez-Pardo D, Puig-Asensio M, Rodriguez V, Ferrer C, Bartolome R, et al. First recurrence of Clostridium difficile infection: clinical relevance, risk factors, and prognosis. European Journal of Clinical Microbiology and Infectious Diseases. 2016;35(3):371-8.

88. Larrainzar-Coghen T, Rodriguez-Pardo D, Fernandez-Hidalgo N, Puig-Asensio M, Pigrau C, Ferrer C, et al. Secular trends in the epidemiology of Clostridium difficile infection (CDI) at a tertiary care hospital in Barcelona, 2006-2015: A prospective observational study. Anaerobe. 2018;51:54-60.

89. Rodriguez-Pardo D, Almirante B, Bartolome RM, Pomar V, Mirelis B, Navarro F, et al. Epidemiology of clostridium difficile infection and risk factors for unfavorable clinical outcomes: Results of a hospital-based study in Barcelona, Spain. Journal of Clinical Microbiology. 2013;51(5):1465-73.

90. Salva S, Duran N, Rodriguez V, Nieto L, Serra J, Rello J, et al. Clostridium difficile in the ICU: study of the incidence, recurrence, clinical characteristics and complications in a university hospital. Med Intensiva. 2014;38(3):140-5.

91. Banks A, Brown DJ, Mather H, Coia JE, Wiuff C. Sentinel community Clostridium difficile infection (CDI) surveillance in Scotland, April 2013 to March 2014. Anaerobe. 2016;37:49-53.

92. Cottom L, Inkster T. Enhanced surveillance of clostridium difficile infection: A reassessment of 2015-2016 reporting in greater Glasgow & Clyde, Scotland. Antimicrobial Resistance and Infection Control. 2017;6(Supplement 3):32.

93. Eyre DW, Walker AS, Wyllie D, Dingle KE, Griffiths D, Finney J, et al. Predictors of first recurrence of clostridium difficile infection: Implications for initial management. Clinical Infectious Diseases. 2012;55(SUPPL.2):S77-S87.

94. Fellmeth G, Yarlagadda S, Iyer S. Epidemiology of community-onset Clostridium difficile infection in a community in the South of England. Journal of Infection and Public Health. 2010;3(3):118-23.

95. Health Protection Scotland. Healthcare Associated Infections. 2018.

96. King A, Mullish BH, Williams HRT, Aylin P. Comparative epidemiology of Clostridium difficile infection: England and the USA. International Journal for Quality in Health Care. 2017;29(6):785-91.

97. Marwick CA, Yu N, Lockhart MC, McGuigan CC, Wiuff C, Davey PG, et al. Community-associated Clostridium difficile infection among older people in Tayside, Scotland, is associated with antibiotic exposure and care home residence: Cohort study with nested case-control. Journal of Antimicrobial Chemotherapy. 2013;68(12):2927-33.

98. Public Health England. Clostridium difficile (C. difficile) infection: annual data. GOV.UK 2018 [Available from: <https://www.gov.uk/government/statistics/clostridium-difficile-infection-annual-data>.

99. Rana F, Kirkley R, Brown S, Nayar D, Dhar A. Clostridium difficile infection in county Durham Hospitals: A changing clinical profile over 5 years. United European Gastroenterology Journal. 2015;3(5 SUPPL. 1):A622-A3.

100. Reddy S, Taori S, Poxton IR. Changes in laboratory and clinical workload for Clostridium difficile infection from 2003 to 2007 in hospitals in Edinburgh. Clinical Microbiology and Infection. 2010;16(4):340-6.

101. Virgincar N, Iyer S, Downing L, Stacey A. Retrospective study of the epidemiology of Clostridium difficile infection in a district general hospital in United Kingdom. Clinical Microbiology and Infection. 2011;17(SUPPL. 4):S579.

102. Wiuff C, Brown DJ, Mather H, Banks AL, Eastaway A, Coia JE. The epidemiology of Clostridium difficile in Scotland. Journal of Infection. 2011;62(4):271-9.

103. Ajayeoba O, Culpepper-Morgan JA, Gorantla S, Lung E. The incidence and severity of clostridium difficile infection: The harlem experience. Gastroenterology. 2013;144(5 SUPPL. 1):S237.

104. Argamany JR, Delgado A, Reveles KR. Clostridium difficile infection health disparities by race among hospitalized adults in the United States, 2001 to 2010. BMC Infectious Diseases. 2016;16(1):454.

105. Armbruster S, Goldkind L. A 5-year retrospective review of experience with Clostridium difficile-associated diarrhea. Military medicine. 2012;177(4):456-9.

106. Clostridium Difficile Hospitalizations, 2011-2015 [Internet]. U.S. Agency for Healthcare Research and Quality. 2018. Available from: [www.hcup-us.ahrq.gov/reports.jsp](file:///\\woksfps01.internal.imsglobal.com\UKHEOR$\01%20Live%20Projects\Ferring\2485657%20-%20SLR,%20NMA,%20economic%20models%20in%20prevention%20of%20rCDI\10%20Dissemination\03%20Manuscript\To%20submit%20to%20BMC%20Infectious%20Diseases\Supplementary%20material\www.hcup-us.ahrq.gov\reports.jsp).

107. Bengualid V, Umesh KC, Alapati J, Berger J. Clostridium difficile at a community hospital in the Bronx, New York: Incidence prevalence and risk factors from 2006 to 2008. American Journal of Infection Control. 2011;39(3):183-7.

108. Benoit SR, McDonald LC, English R, Tokars JI. Automated surveillance of Clostridium difficile infections using biosense. Infection Control and Hospital Epidemiology. 2011;32(1):26-33.

109. Bradley ES, Wu X, Howe E, Haran JP. Medication risk factors for recurrent clostridium difficile infection among the elderly. Academic Emergency Medicine. 2017;24(Supplement 1):S160.

110. Brown KA, Jones M, Adler F, Leecaster M, Nechodom K, Stevens V, et al. The determinants of C. Difficile infection in long-term care facilities: A portrait of patient-and facility-level factors across 90 care regions in the veterans affairs health care system. Antimicrobial Resistance and Infection Control. 2015;4(SUPPL. 1):O36.

111. Brown KA, Jones M, Daneman N, Adler FR, Stevens V, Nechodom KE, et al. Importation, antibiotics, and clostridium difficile infection in veteran long-term care: A multilevel case-control study. Annals of Internal Medicine. 2016;164(12):787-94.

112. Brown KA, Daneman N, Jones M, Nechodom K, Stevens V, Adler FR, et al. Te drivers of acute and long-term care clostridium dificile infection rates: A retrospective multilevel cohort study of 251 facilities. Clinical Infectious Diseases. 2017;65(8):1282-8.

113. Cadena J, Thompson GR, Patterson JE, Nakashima B, Owens A, Echevarria K, et al. Clinical predictors and risk factors for relapsing clostridium difficile infection. American Journal of the Medical Sciences. 2010;339(4):350-5.

114. Campbell RJ, Giljahn L, Machesky K, Katie CW, Lane LM, Porter K, et al. Clostridium difficile infection in Ohio hospitals and nursing homes during 2006. Infection Control and Hospital Epidemiology. 2009;30(6):526-33.

115. Chitnis AS, Holzbauer SM, Belflower RM, Winston LG, Bamberg WM, Lyons C, et al. Epidemiology of community-associated Clostridium difficile infection, 2009 through 2011. JAMA Internal Medicine. 2013;173(14):1359-67.

116. Dean B, Campbell RS, Nathanson BH, Haidar T, Strauss ME, Thomas S. Risk factors associated with Hospital-origin versus communityorigin Clostridium difficile-associated diarrhea. Pharmacotherapy. 2012;32(10):e203-e4.

117. Delate T, Albrecht G, Won K, Jackson A. Ambulatory-treated Clostridium difficile infection: A comparison of community-acquired vs. nosocomial infection. Epidemiology and Infection. 2015;143(6):1225-35.

118. Dubberke ER, McMullen KM, Mayfield JL, Reske KA, Georgantopoulos P, Warren DK, et al. Hospital-associated Clostridium difficile infection: Is it necessary to track community-onset disease? Infection Control and Hospital Epidemiology. 2009;30(4):332-7.

119. Dubberke ER, Butler AM, Hota B, Khan YM, Mangino JE, Mayer J, et al. Multicenter study of the impact of community-onset clostridium difficile infection on surveillance for C. difficile infection. Infection Control and Hospital Epidemiology. 2009;30(6):518-25.

120. Dubberke ER, Butler AM, Yokoe DS, Mayer J, Hota B, Mangino JE, et al. Multicenter study of Clostridium difficile infection rates from 2000 to 2006. Infection Control and Hospital Epidemiology. 2010;31(10):1030-7.

121. Dubberke ER, Butler AM, Nyazee HA, Reske KA, Yokoe DS, Mayer J, et al. The impact of ICD-9-CM code rank order on the estimated prevalence of clostridium difficile infections. Clinical Infectious Diseases. 2011;53(1):20-5.

122. Dubberke ER, Olsen MA, Stwalley D, Kelly CP, Gerding DN, Young-Xu Y, et al. Identification of medicare recipients at highest risk for clostridium difficile infection in the US by population attributable risk analysis. PLoS ONE. 2016;11(2):e0146822.

123. Dumyati G, Stevens V, Hannett GE, Thompson AD, Long C, Maccannell D, et al. Community-associated Clostridium difficile infections, Monroe county, New York, USA. Emerging Infectious Diseases. 2012;18(3):392-400.

124. Evans ME, Simbartl LA, Kralovic SM, Jain R, Roselle GA. Clostridium difficile infections in veterans health administration acute care facilities. Infection Control and Hospital Epidemiology. 2014;35(8):1037-42.

125. Freedberg DE, Salmasian H, Friedman C, Abrams JA. Proton pump inhibitors and risk for recurrent Clostridium difficile infection among inpatients. Am J Gastroenterol. 2013;108(11):1794-801.

126. Friedman H, Navaratnam P, Reardon G, High KP, Strauss M. Characteristics and outcomes related to clostridium difficile-associated disease in us long term care facilities. Journal of the American Geriatrics Society. 2012;60(SUPPL. 4):S229.

127. Garg S, Mirza YR, Girotra M, Kumar V, Yoselevitz S, Segon A, et al. Epidemiology of Clostridium difficile-associated disease (CDAD): A shift from hospital-acquired infection to long-term care facility-based infection. Digestive Diseases and Sciences. 2013;58(12):3407-12.

128. Guerrero DM, Nerandzic MM, Jury LA, Chang S, Jump RL, Donskey CJ. Clostridium difficile infection in a department of veterans affairs long-term care facility. Infection Control and Hospital Epidemiology. 2011;32(5):513-5.

129. Haran JP, Bradley E, Howe E, Wu X, Tjia J. Medication Exposure and Risk of Recurrent Clostridium difficile Infection in Community-Dwelling Older People and Nursing Home Residents. Journal of the American Geriatrics Society. 2018;66(2):333-8.

130. Hebert C, Du H, Peterson LR, Robicsek A. Electronic health record-based detection of risk factors for clostridium difficile infection relapse. Infection Control and Hospital Epidemiology. 2013;34(4):407-14.

131. Hudspeth WB, Qeadan F, Phipps EC. Disparities in the incidence of community-acquired Clostridioides difficile infection: An area-based assessment of the role of social determinants in Bernalillo County, New Mexico. American Journal of Infection Control. 2019;47(7):773-9.

132. Hunter JC, Mu Y, Dumyati GK, Farley MM, Winston LG, Johnston HL, et al. Burden of Nursing Home-Onset Clostridium difficile Infection in the United States: Estimates of Incidence and Patient Outcomes. Open Forum Infect Dis. 2016;3(1):ofv196.

133. Ihimoyan A, Nayudu SK, Sinha A, Makker J, Majeed S, Blum S, et al. National hospital quality measures and clostridium difficile infection in an inner city hospital. American Journal of Gastroenterology. 2010;105(SUPPL. 1):S147-S8.

134. Jacob J, Wu J, Han J, Nelson DB. Clostridium difficile in an Urban, University-Affiliated Long-Term Acute-Care Hospital. Infection Control and Hospital Epidemiology. 2017;38(3):294-9.

135. Jarvis WR, Schlosser J, Jarvis AA, Chinn RY. National point prevalence of Clostridium difficile in US health care facility inpatients, 2008. American Journal of Infection Control. 2009;37(4):263-70.

136. Kanamori H, Weber DJ, Dibiase LM, Sickbert-Bennett EE, Brooks R, Teal L, et al. Longitudinal trends in all healthcare-associated infections through comprehensive hospital-wide surveillance and infection control measures over the past 12 years: Substantial burden of healthcare-associated infections outside of intensive care units and "other" types of infection. Infection Control and Hospital Epidemiology. 2015;36(10):1139-47.

137. Kang J, Sickbert-Bennett EE, Brown VM, Weber DJ, Rutala WA. Changes in the incidence of health care-associated pathogens at a university hospital from 2005 to 2011. American Journal of Infection Control. 2014;42(7):770-5.

138. Karanika S, Grigoras C, Flokas ME, Alevizakos M, Kinamon T, Kojic EM, et al. The Attributable Burden of Clostridium difficile Infection to Long-Term Care Facilities Stay: A Clinical Study. Journal of the American Geriatrics Society. 2017;65(8):1733-40.

139. Khanna S, Pardi DS, Aronson SL, Kammer PP, Orenstein R, St Sauver JL, et al. The epidemiology of community-acquired Clostridium difficile infection: a population-based study. The American journal of gastroenterology. 2012;107(1):89-95.

140. Khanna S, Hernandez MJR, Mara K, Harmsen WS, Pardi D. Changing epidemiology of clostridium difficile from predominantly hospital-acquired to community-acquired infection. Gastroenterology. 2016;150(4 SUPPL. 1):S832-S3.

141. Khanna S, Gupta A, Baddour LM, Pardi DS. Epidemiology, outcomes, and predictors of mortality in hospitalized adults with Clostridium difficile infection. Internal and emergency medicine. 2016;11(5):657-65.

142. Kim JH, Toy D, Muder RR. Clostridium difficile infection in a long-term care facility: Hospital-associated illness compared with long-term care-associated illness. Infection Control and Hospital Epidemiology. 2011;32(7):656-60.

143. King RN, Lager SL. Incidence of Clostridium difficile infections in patients receiving antimicrobial and acid-suppression therapy. Pharmacotherapy. 2011;31(7):642-8.

144. Kroner PT, Corral JE, D'Assuncao M, Gutierrez JP, Schwingel G, Jovanovic I, et al. Shifting patterns of clostridium difficile seasonal variations in the past decade across the United States. American Journal of Gastroenterology. 2017;112(Supplement 1):S604.

145. Kuntz JL, Chrischilles EA, Pendergast JF, Herwaldt LA, Polgreen PM. Incidence of and risk factors for community-associated Clostridium difficile infection: A nested case-control study. BMC Infectious Diseases. 2011;11:194.

146. Kuntz JL, Johnson ES, Raebel MA, Petrik AF, Yang X, Glenn K, et al. The epidemiology of clostridium difficile infections at kaiser permanente. Pharmacoepidemiology and Drug Safety. 2011;20(SUPPL. 1):S179.

147. Kuntz JL, Johnson ES, Raebel MA, Petrik AF, Yang X, Thorp ML, et al. Epidemiology and healthcare costs of incident clostridium difficile infections identified in the outpatient healthcare setting. Infection Control and Hospital Epidemiology. 2012;33(10):1031-8.

148. Kutty PK, Woods CW, Sena AC, Benoit SR, Naggie S, Frederick J, et al. Risk factors for and estimated incidence of community-associated Clostridium difficile infection, North Carolina, USA. Emerging Infectious Diseases. 2010;16(2):197-204.

149. Lessa FC, Mu Y, Winston LG, Dumyati GK, Farley MM, Beldavs ZG, et al. Determinants of Clostridium difficile infection incidence across diverse United States geographic locations. Open Forum Infectious Diseases. 2014;1(2):ofu048.

150. Lessa FC, Mu Y, Bamberg WM, Beldavs ZG, Dumyati GK, Dunn JR, et al. Burden of Clostridium difficile infection in the United States. New England Journal of Medicine. 2015;372(9):825-34.

151. Luo R, Barlam TF. Ten-year review of Clostridium difficile infection in acute care hospitals in the USA, 2005-2014. Journal of Hospital Infection. 2018;98(1):40-3.

152. Ma GK, Brensinger CM, Wu Q, Lewis JD. Increasing incidence of multiply recurrent Clostridium difficile infection in the United States: A cohort study. Annals of Internal Medicine. 2017;167(3):152-8.

153. McNabb-Baltar J, Yaghoobi M, Trinh QD. Clostridium difficile infection emergency department visits in the united states: Patterns of care, admission and mortality. Gastroenterology. 2013;144(5 SUPPL. 1):S239-S40.

154. McNabb-Baltar J, Yaghoobi M, O'Byrne M, Soulellis CA, Trinh QD. Burden of clostridium difficile-associated hospitalizations in the united states. Gastroenterology. 2013;144(5 SUPPL. 1):S239.

155. Miller AC, Polgreen LA, Cavanaugh JE, Polgreen PM. Hospital Clostridium difficile infection (CDI) incidence as a risk factor for hospital-associated CDI. American Journal of Infection Control. 2016;44(7):825-9.

156. Murphy CR, Avery TR, Dubberke ER, Huang SS. Frequent hospital readmissions for clostridium difficile infection and the impact on estimates of hospital-associated c. difficile burden. Infection Control and Hospital Epidemiology. 2012;33(1):20-8.

157. Naggie S, Frederick J, Pien BC, Miller BA, Provenzale DT, Goldberg KC, et al. Community-associated Clostridium difficile infection: Experience of a veteran affairs medical center in southeastern USA. Infection. 2010;38(4):297-300.

158. Naik S, Giddings S, Kottoor R, Li X. Risk factors for community acquired vs hospital acquired clostridium difficile infection. Gastroenterology. 2012;142(5 SUPPL. 1):S680.

159. Olsen MA, Young-Xu Y, Stwalley D, Kelly CP, Gerding DN, Saeed MJ, et al. The burden of clostridium difficile infection: Estimates of the incidence of CDI from U.S. Administrative databases. BMC Infectious Diseases. 2016;16(1):177.

160. Olson B, Floyd RA, Howard J, Hassanein T, Warm K, Oen R. A multipronged approach to decrease the risk of Clostridium difficile infection at a community hospital and long-Term care facility. Journal of Clinical Outcomes Management. 2015;22(9):398-406.

161. Pakyz AL, Jawahar R, Wang Q, Harpe SE. Medication risk factors associated with healthcare-associated Clostridium difficile infection: A multilevel model case-control study among 64 US academic medical centres. Journal of Antimicrobial Chemotherapy. 2014;69(4):1127-31.

162. Pant C, Sferra TJ, Olyaee M, Gilroy R, Anderson MP, Rastogi A, et al. Emergency department visits related to Clostridium difficile infection: results from the nationwide emergency department sample, 2006 through 2010. Academic emergency medicine : official journal of the Society for Academic Emergency Medicine. 2015;22(1):117-9.

163. Parasa S, Kennedy KF, Sharma P. Readmissions after hospitalization for clostridium difficile infection in the United States: Economic burden, epidemiology and risk factors. Gastroenterology. 2017;152(5 Supplement 1):S347.

164. Pawar D, Tsay R, Nelson DS, Elumalai MK, Lessa FC, Clifford McDonald L, et al. Burden of clostridium difficile infection in Long-term care facilities in Monroe County, New York. Infection Control and Hospital Epidemiology. 2012;33(11):1107-12.

165. Pechal A, Lin K, Allen S, Reveles K. National age group trends in Clostridium difficile infection incidence and health outcomes in United States Community Hospitals. BMC Infectious Diseases. 2016;16(1):682.

166. Press A, Ku B, McCullagh L, Rosen L, Richardson S, McGinn T. Developing a clinical predication rule for first onset hospital-acquired c. difficile infections. Journal of General Internal Medicine. 2016;31(2 SUPPL. 1):S180-S1.

167. Reeves JS, Evans ME, Simbartl LA, Kralovic SM, Kelly AA, Jain R, et al. Clostridium difficile infections in veterans health administration long-term care facilities. Infection Control and Hospital Epidemiology. 2016;37(3):295-300.

168. Reveles KR, Lee GC, Boyd NK, Frei CR. The rise in Clostridium difficile infection incidence among hospitalized adults in the United States: 2001-2010. American Journal of Infection Control. 2014;42(10):1028-32.

169. Reveles KR, Lawson KA, Mortensen EM, Pugh MJV, Koeller JM, Argamany JR, et al. National epidemiology of initial and recurrent Clostridium difficile infection in the Veterans Health Administration from 2003 to 2014. PLoS ONE. 2017;12(12):e0189227.

170. Reveles KR, Pugh MJV, Lawson KA, Mortensen EM, Koeller JM, Argamany JR, et al. Shift to community-onset Clostridium difficile infection in the national Veterans Health Administration, 2003-2014. American Journal of Infection Control. 2018;46(4):431-5.

171. Saffouri G, Khanna S, Heien H, Sangaralingham L, Pardi DS. Prevalence and recurrence of clostridium difficile infection among a commercially insured population: 2005 and 2014. Gastroenterology. 2017;152(5 Supplement 1):S341.

172. Shivashankar R, Khanna S, Kammer P, Harmsen W, Zinsmeister A, Baddour L, et al. Clinical predictors of recurrent clostridium difficile infection presidential poster. American Journal of Gastroenterology. 2012;107(SUPPL. 1):S213.

173. Smith AM, Wuerth BA, Wiemken TL, Arnold FW. Prevalence of Clostridium difficile infection presenting to US EDs. American Journal of Emergency Medicine. 2015;33(2):238-43.

174. Troppy TS, Mishra T, Barton K, Caten E, Vo Q, McHale E, et al. Using public health surveillance data to measure Clostridium difficile infection population burden in Massachusetts. American Journal of Infection Control. 2019;47(2):211-2.

175. Verlee KE, Finks JL, Wilkins MJ, Wells EV. Michigan Clostridium difficile hospital discharges: Frequency, mortality, and charges, 2002-2008. Public Health Reports. 2012;127(1):62-71.

176. Watson T, Hickok J, Fraker S, Korwek K, Poland RE, Septimus E. Evaluating the Risk Factors for Hospital-Onset Clostridium difficile Infections in a Large Healthcare System. Clinical Infectious Diseases. 2018;66(12):1957-9.

177. Winkler KR, Burgess DR, Dasher T, Burgess DS. Evaluation of the management of Clostrium difficile infection in hospitalized adult patients. Pharmacotherapy. 2010;30(10):409e.

178. Young-Xu Y, Kuntz JL, Gerding DN, Neily J, Mills P, Dubberke ER, et al. Clostridium difficile infection among veterans health administration patients. Infection Control and Hospital Epidemiology. 2015;36(9):1038-45.

179. Yu H, Baser O, Wang L. Burden of Clostridium difficile-associated disease among patients residing in nursing homes: a population-based cohort study. BMC geriatrics. 2016;16(1):193.

180. Zarowitz BJ, Allen C, O'Shea T, Strauss ME. Risk factors, clinical characteristics, and treatment differences between residents with and without nursing home-and non-nursing home-acquired Clostridium difficile infection. Journal of Managed Care Pharmacy. 2015;21(7):585-95.

181. Ziakas PD, Joyce N, Zacharioudakis IM, Zervou FN, Besdine RW, Mor V, et al. Prevalence and impact of Clostridium difficile infection in elderly residents of long-term care facilities, 2011: A nationwide study. Medicine (United States). 2016;95(31):e4187.

182. Zilberberg M, Tillotson G, Kollef M, Shorr A. Update on clostridium difficile hospitalizations in the US through 2008: The epidemic continues. Chest Conference: CHEST. 2011;140(4 (Suppl.)):269A.

183. Zilberberg MD, Tabak YP, Sievert DM, Derby KG, Johannes RS, Sun X, et al. Using electronic health information to risk-stratify rates of Clostridium difficile infection in US hospitals. Infection Control and Hospital Epidemiology. 2011;32(7):649-55.

184. Zilberberg MD, Reske K, Olsen M, Yan Y, Dubberke ER. Risk factors for recurrent Clostridium difficile infection (CDI) hospitalization among hospitalized patients with an initial CDI episode: a retrospective cohort study. BMC infectious diseases. 2014;14:306.

185. Zilberberg MD, Shorr AF, Jesdale WM, Tjia J, Lapane K. Recurrent Clostridium difficile infection among Medicare patients in nursing homes: A population-based cohort study. Medicine (Baltimore). 2017;96(10):e6231.
